# Supplementary figures and images for: Long transposon-rich centromeres in an oomycete reveal divergence of centromere features in Stramenopila-Alveolata-Rhizaria lineages
Source: PLoS Genet. 2020 Mar 9;16(3):e1008646. doi: 10.1371/journal.pgen.1008646 (PMC7082073; doi:10.1371/journal.pgen.1008646)

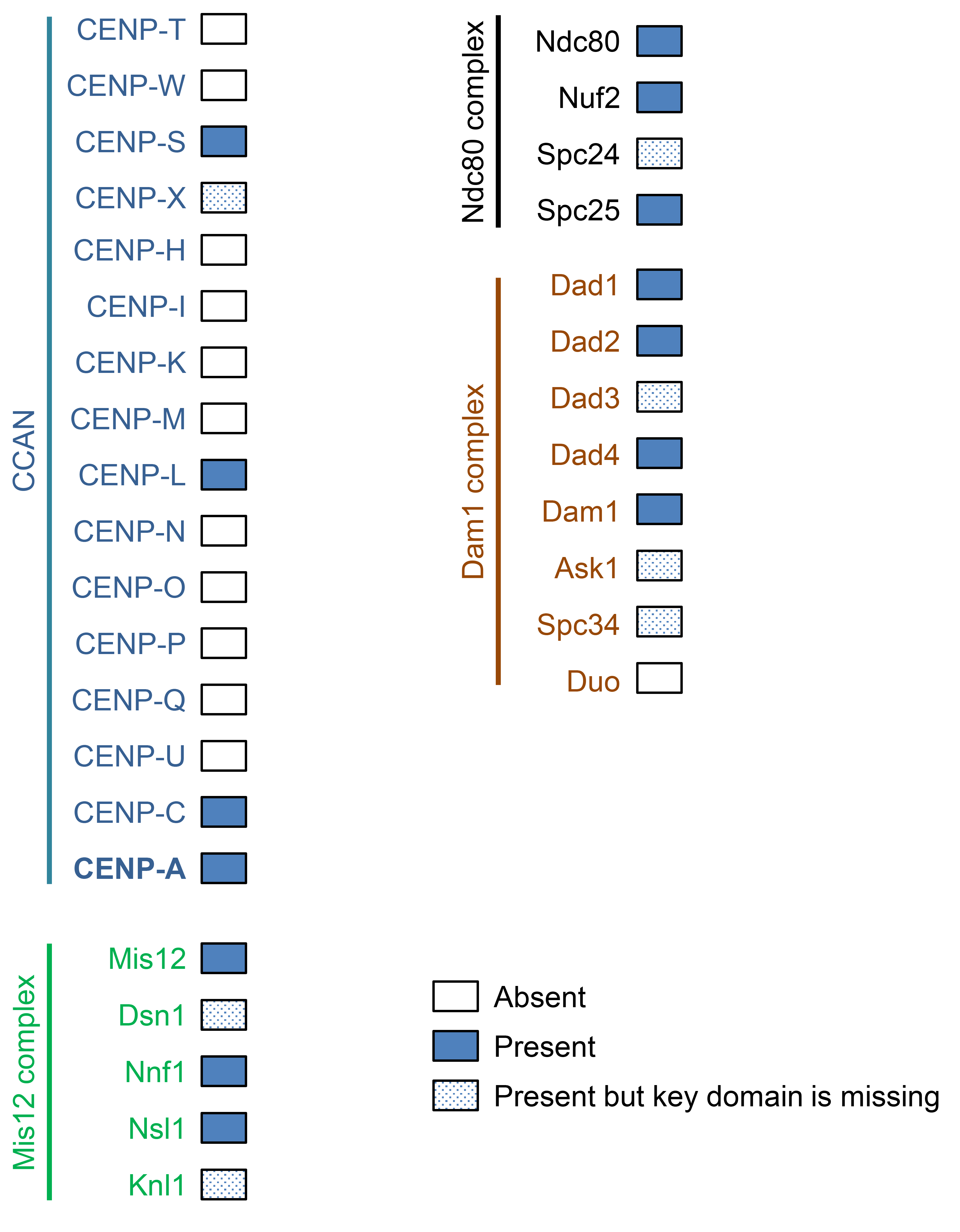

Supplement: S1 Fig — Kinetochore orthologs were identified based on BLAST searches. P. sojae CENP-A (in bold) was selected to track subcellular localization of centromere/kinetochore and profile centromere sequences. Sequences of P. sojae putative core kinetochore proteins are listed in S1 File. (TIF) [file pgen.1008646.s001.tif]

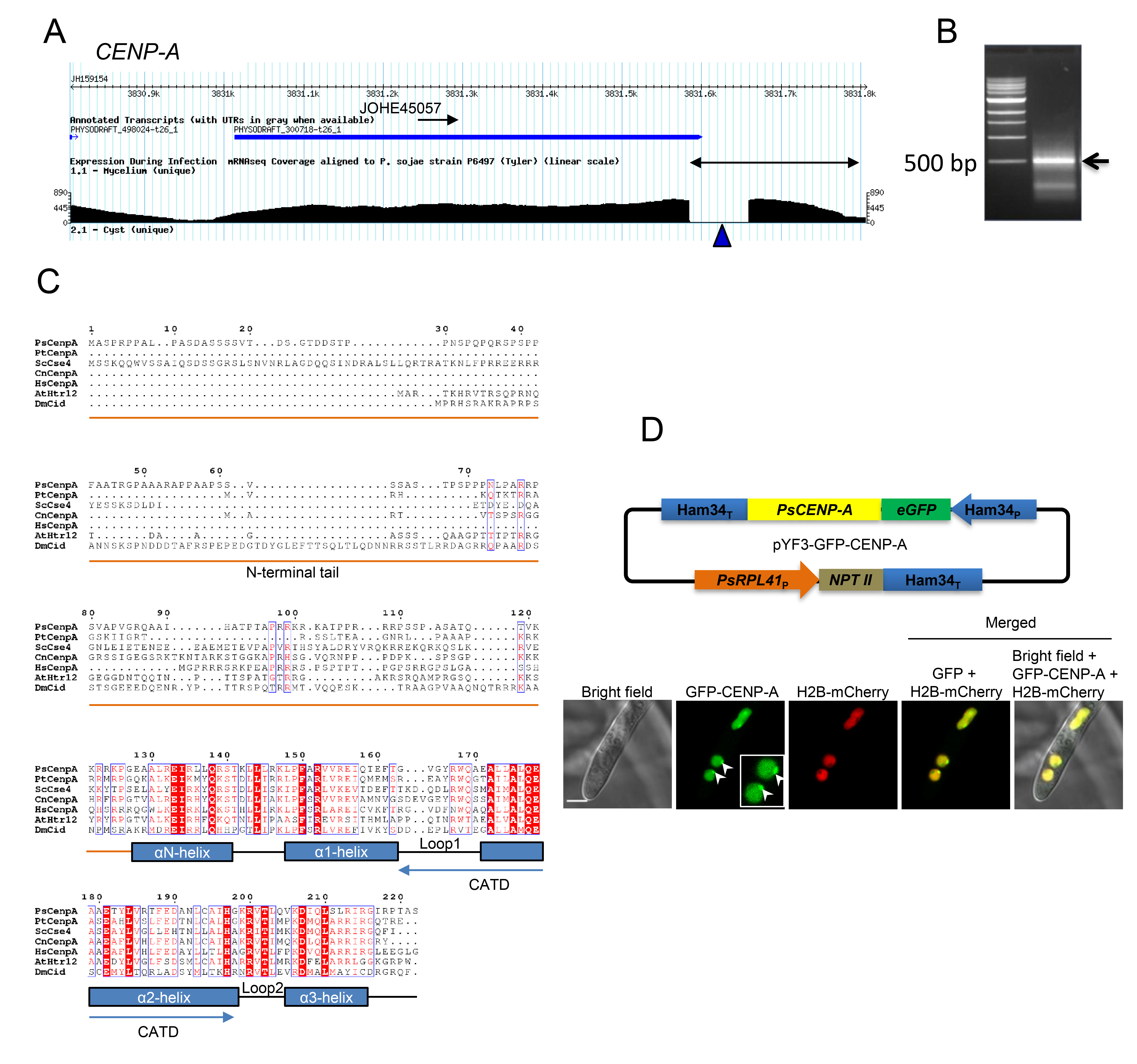

Supplement: S2 Fig — (A) Image of CENP-A gene model and RNA-seq log scale coverage taken from FungiDB. The left right arrow and triangle denote an erroneous P. sojae CENP-A gene model caused by an intron that was missed in the gene model prediction. (B) Electrophoresis image showing 3’-RACE result of CENP-A. 5’-primer JOHE45057 (not shown in scale) served as a gene-specific primer for 3’-RACE (See S6 Table). (C) Alignment of P. sojae CENP-A with orthologs from different organisms. Ps, P. sojae; Pt, Phaeodactylum tricornutum (diatom); Sc, Saccharomyces cerevisiae; Cn, Cryptococcus neoformans; Hs, Homo sapiens; At, Arabidopsis thaliana; Dm, Drosophila melanogaster. (D) Transient expression of GFP tagged CENP-A in P. sojae transformants. Upper panel, a plasmid constructed for transient expression of CENP-A. Expression of P. sojae CENP-A (PsCENP-A) is driven by a constitutive promoter derived from the B. lactucae HAM34 gene. Lower panel, subcellular localization of GFP-tagged CENP-A in the P. sojae transformants based on the constructs shown in the upper panel. (TIF) [file pgen.1008646.s002.tif]

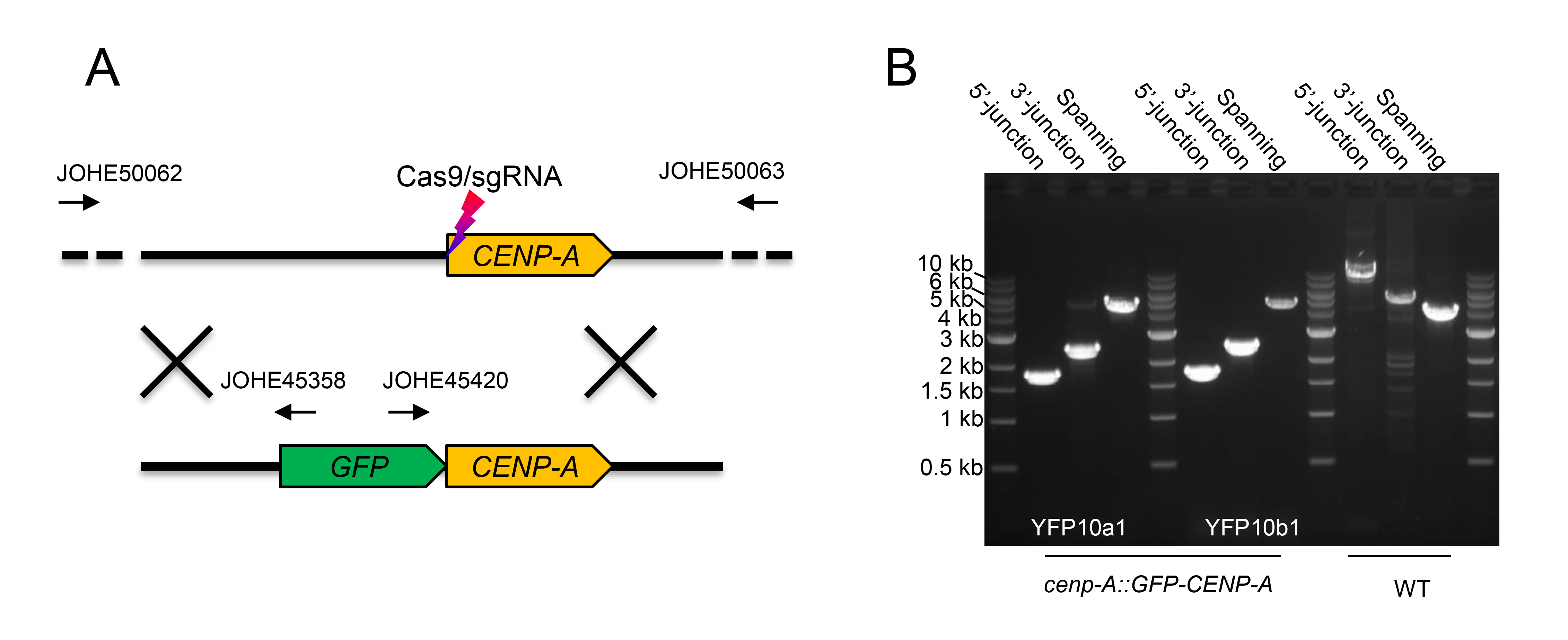

Supplement: S3 Fig — (A) Schematic of gene replacement of the endogenous CENP-A with GFP-CENP-A. Lightning bolts, an sgRNA guide sequence was designed overlapping the start codon of CENP-A. Primer pairs, JOHE50062/JOHE45358, JOHE45420/JOHE50063, JOHE50062/JOHE50063 were used for 5’-junction, 3’-junction, and spanning diagnostic PCR screening GFP-CENP-A mutants. See S6 Table for the primer information. (B) Representative genotyping results of zoospore isolated (homokaryotic) GFP-CENP-A strains (YFP10a1 and YFP10b1, see S5 Table for their genetic backgrounds). Products observed in the 5’- and 3’-junction PCR of wild type (WT) are non-specific amplicons. (TIF) [file pgen.1008646.s003.tif]

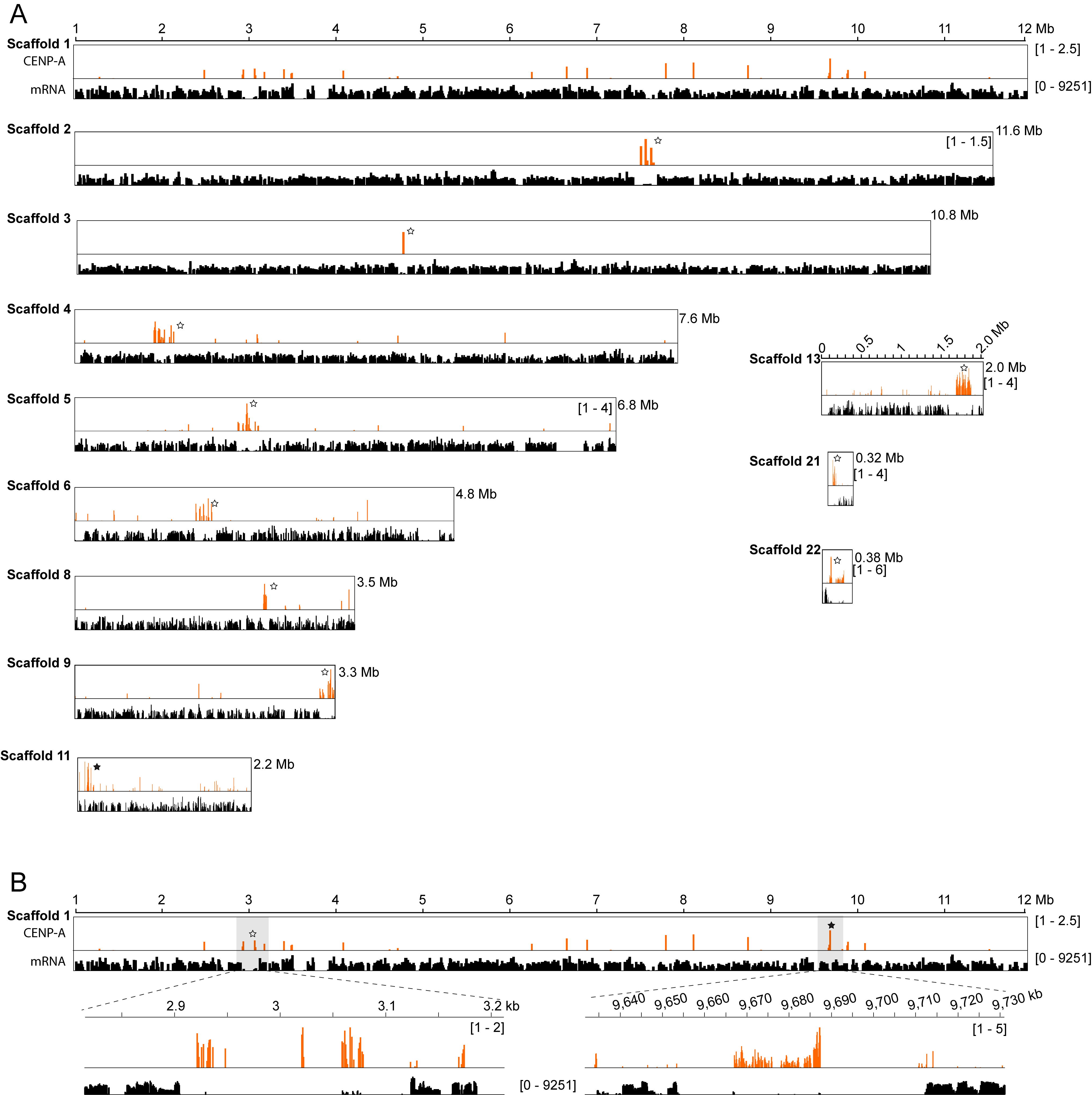

Supplement: S4 Fig — (A) CENP-A enrichment identified in 12 scaffolds. Solid and hollow stars denote CENP-A enrichment regions that are sequence-gap free or contain gaps, respectively. All CENP-A profiles shown have been normalized to input. mRNA profiles are shown as log-scales. (B) Two putative centromeric regions in Scaffold 1 were identified by poor transcription and the syntenic regions in the Psojae2019.1 assembly. (TIF) [file pgen.1008646.s004.tif]

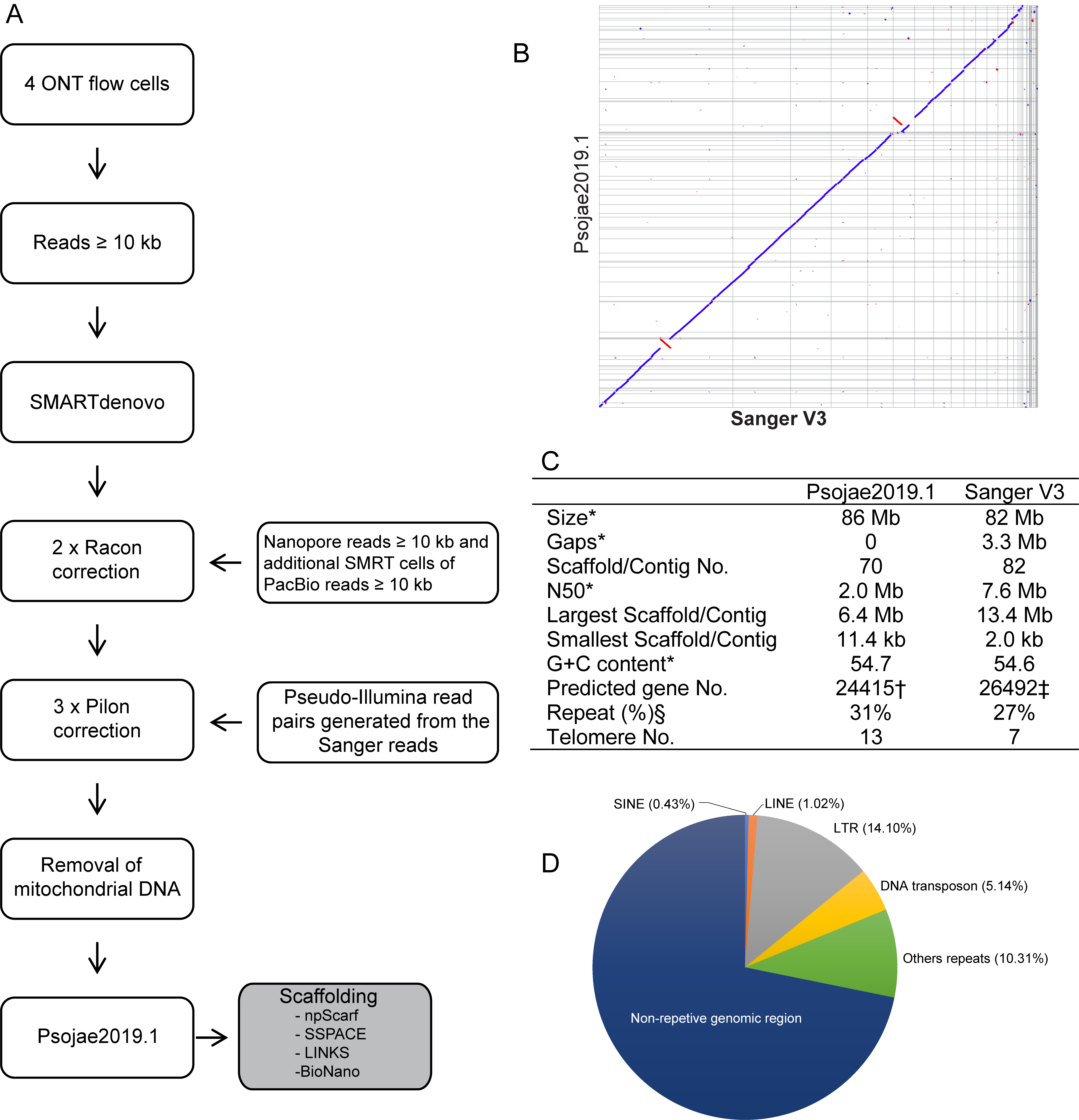

Supplement: S5 Fig — (A) Pipeline used to generate the assembly of Psojae2019.1. Box in grey, different scaffolding programs were employed to enhance the contiguity of the assembly (See more details in S10 Fig and S3 Table). As some of them generated conflict and sequence gaps, we opted to use the contig-level assembly for the centromere study. (B) Dotplot comparison of the long-read Nanopore assembly Psojae2019.1 against the Sanger assembly. (C) Genome assembly metrics. *Statistics for both the Psojae2019.1 and Sanger V3 assemblies were calculated by QUAST [89]; †Annotation based on the repeat-masked assembly (See Method). ‡Annotation obtained from FungiDB release 33 (https://fungidb.org/fungidb/). §Measured by RepeatModeler (See Methods). (D) Pie chart summarizing retroelements (LINE, SINE and LTR), DNA transposons and other repeat sequences predicted in the Psoaje2019.1 assembly. (TIF) [file pgen.1008646.s005.tif]

A

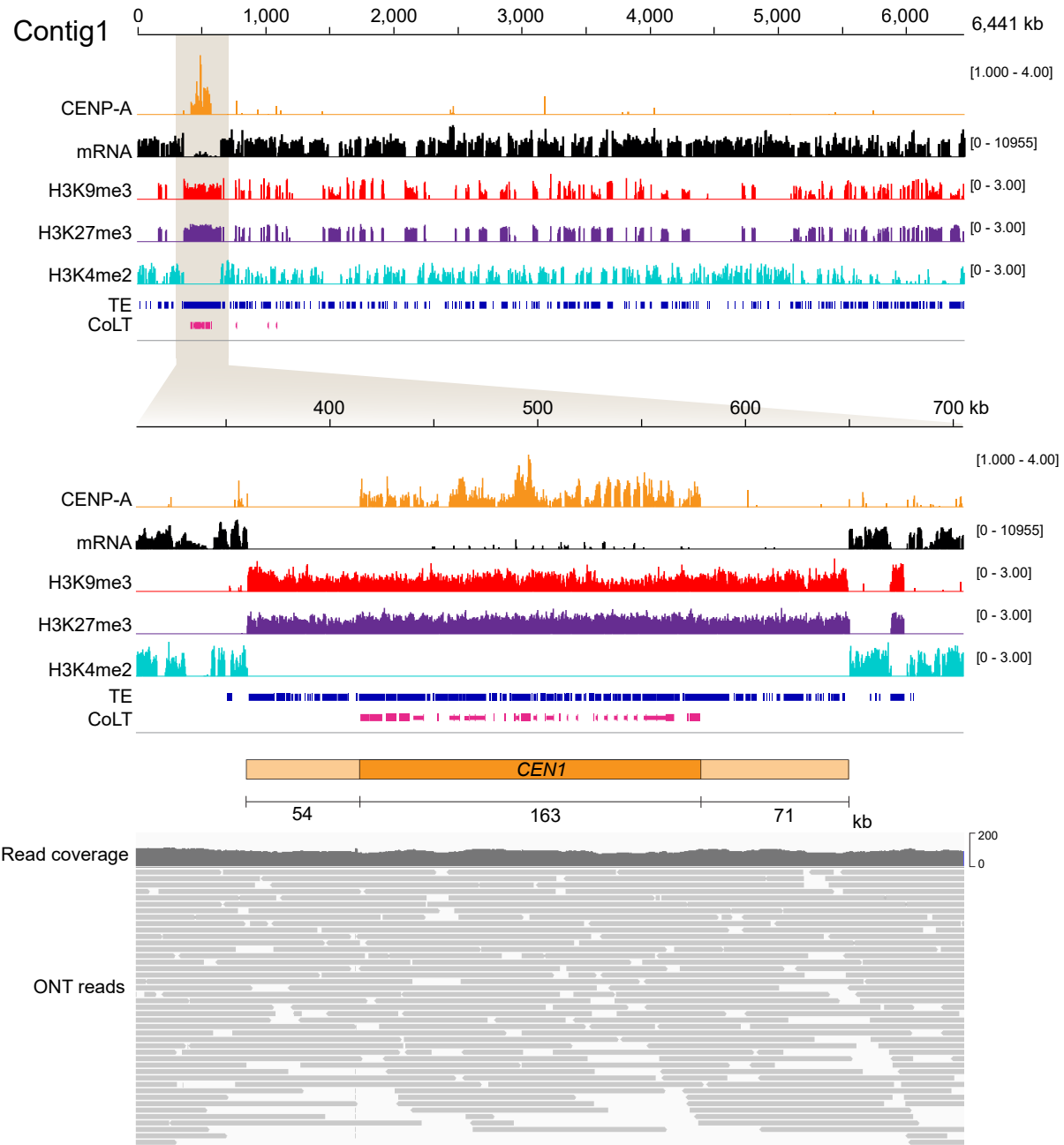

B

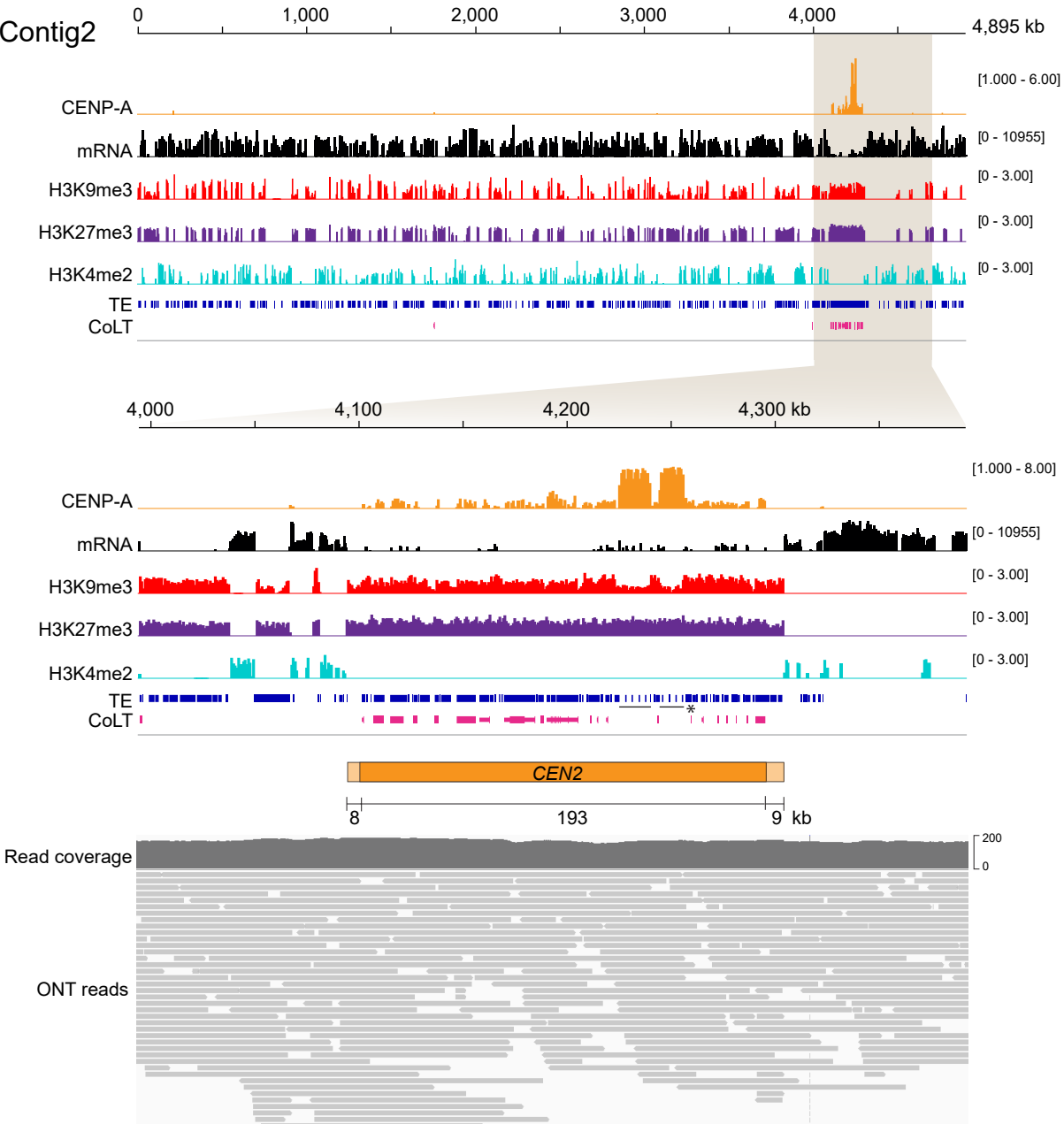

C

Contig3

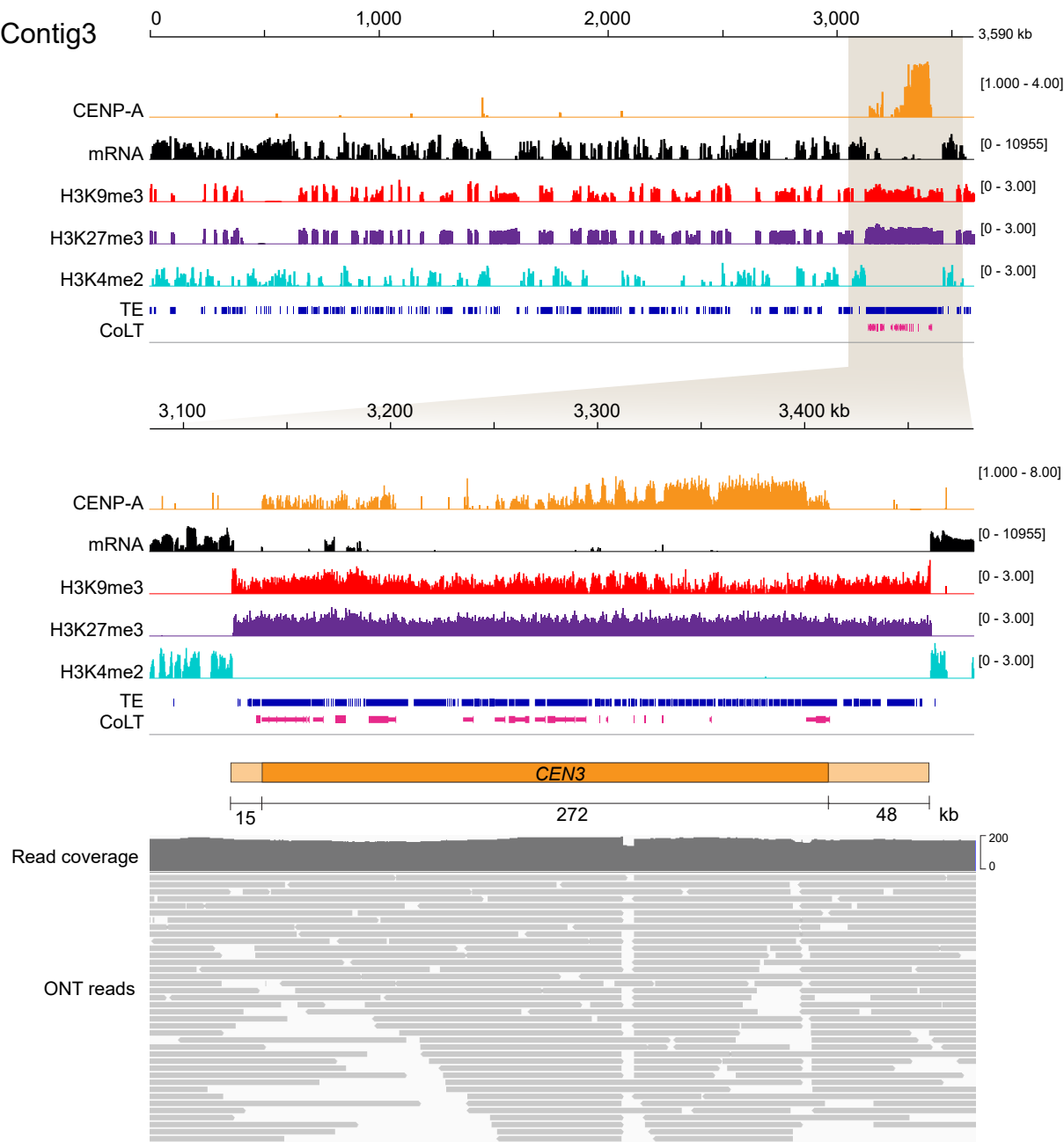

D

Contig11

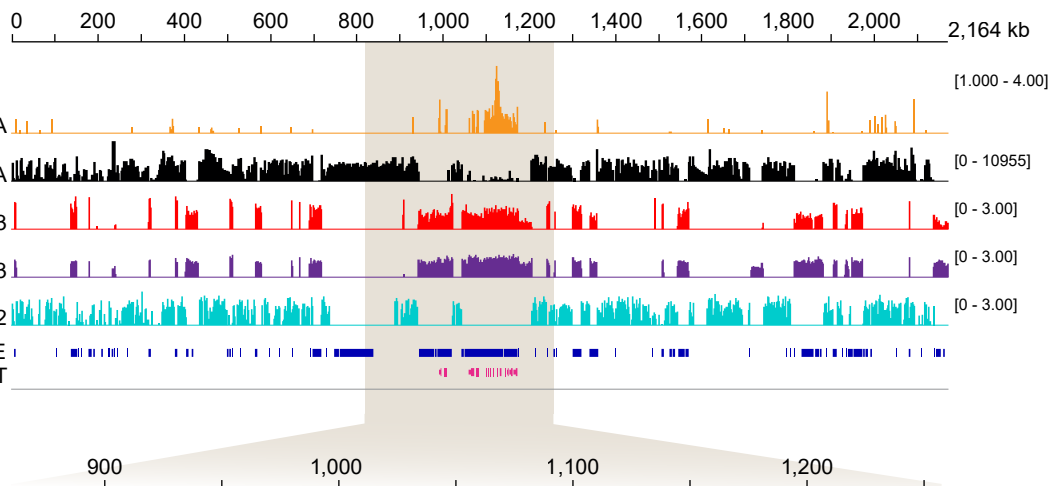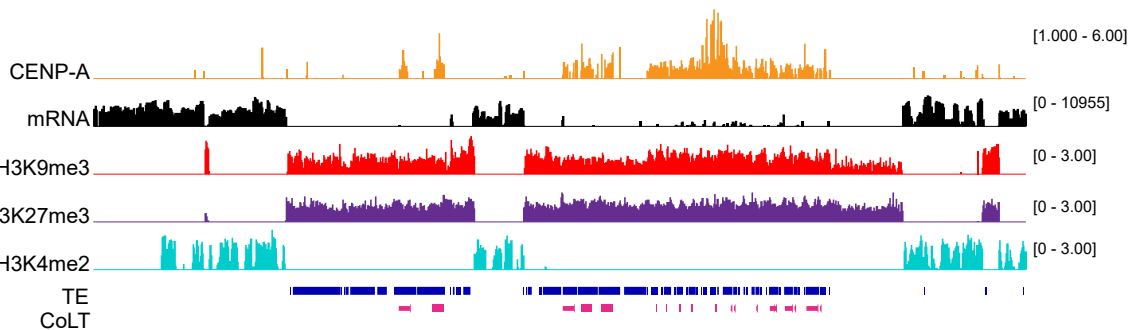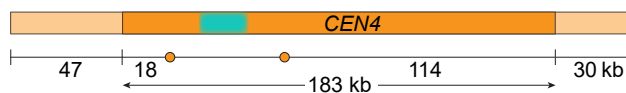

Read coverage

ONT reads

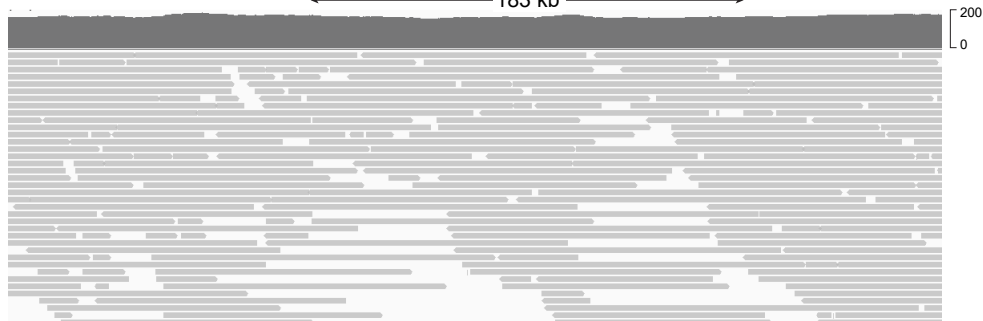

E

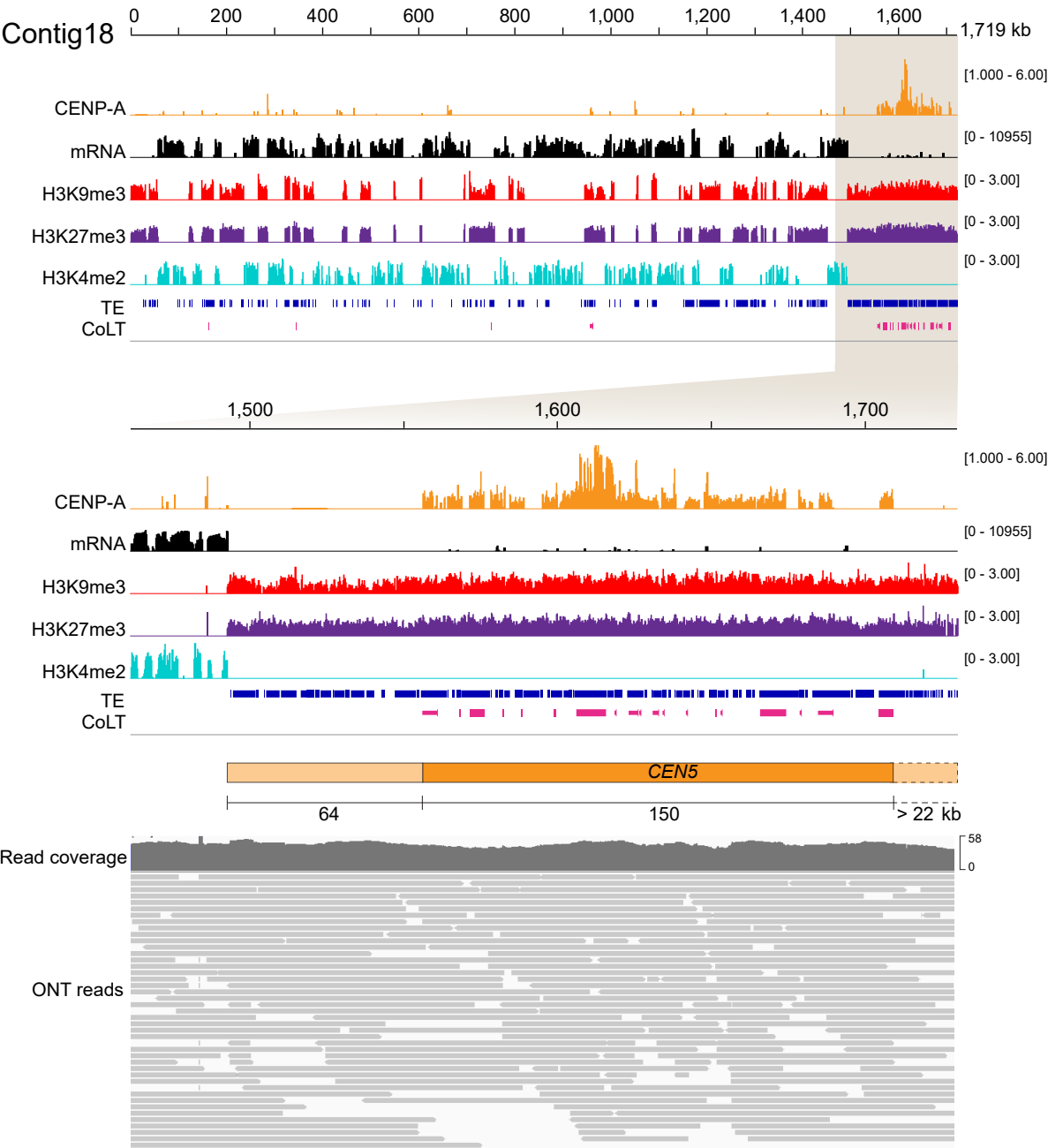

F

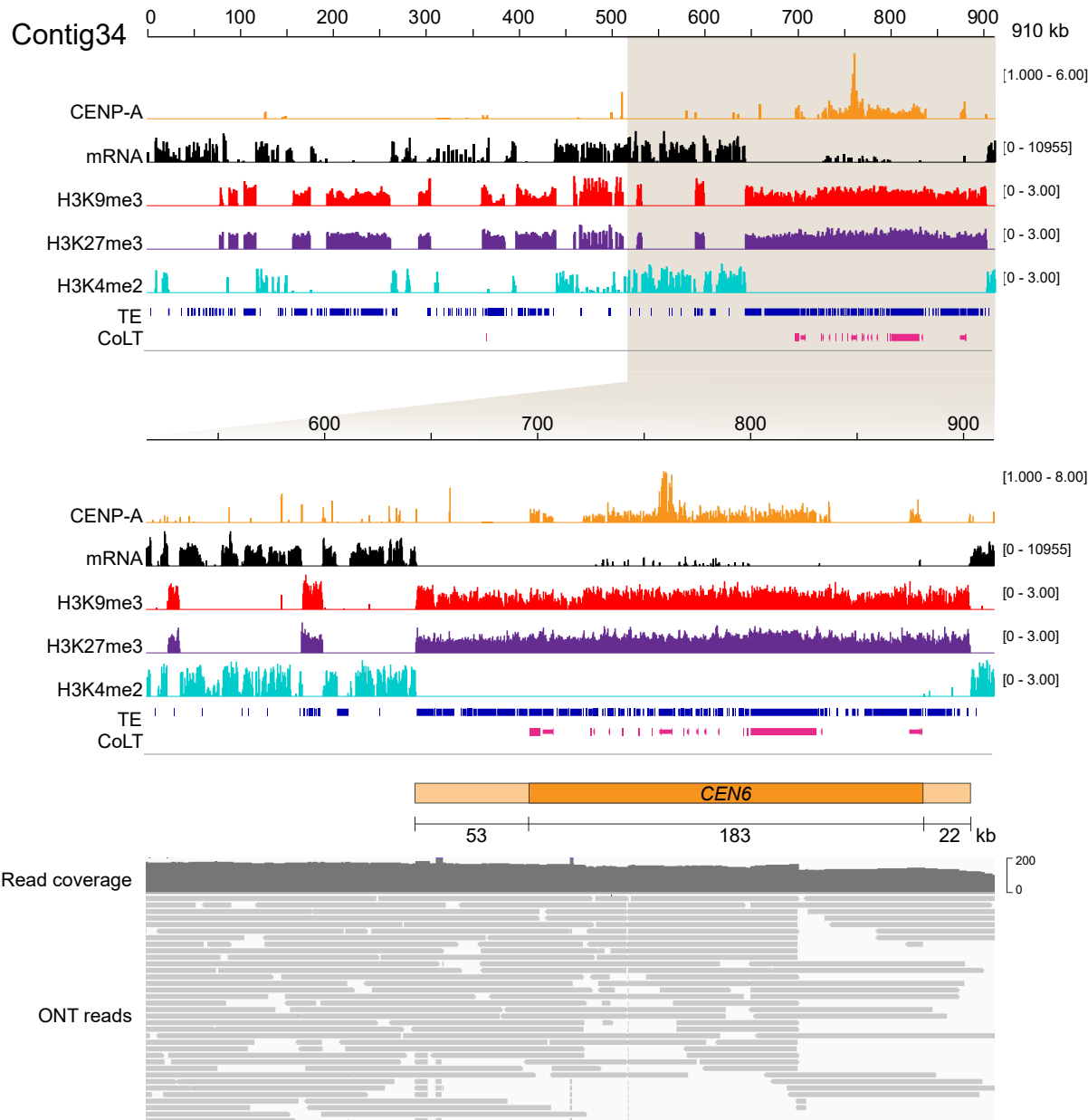

G

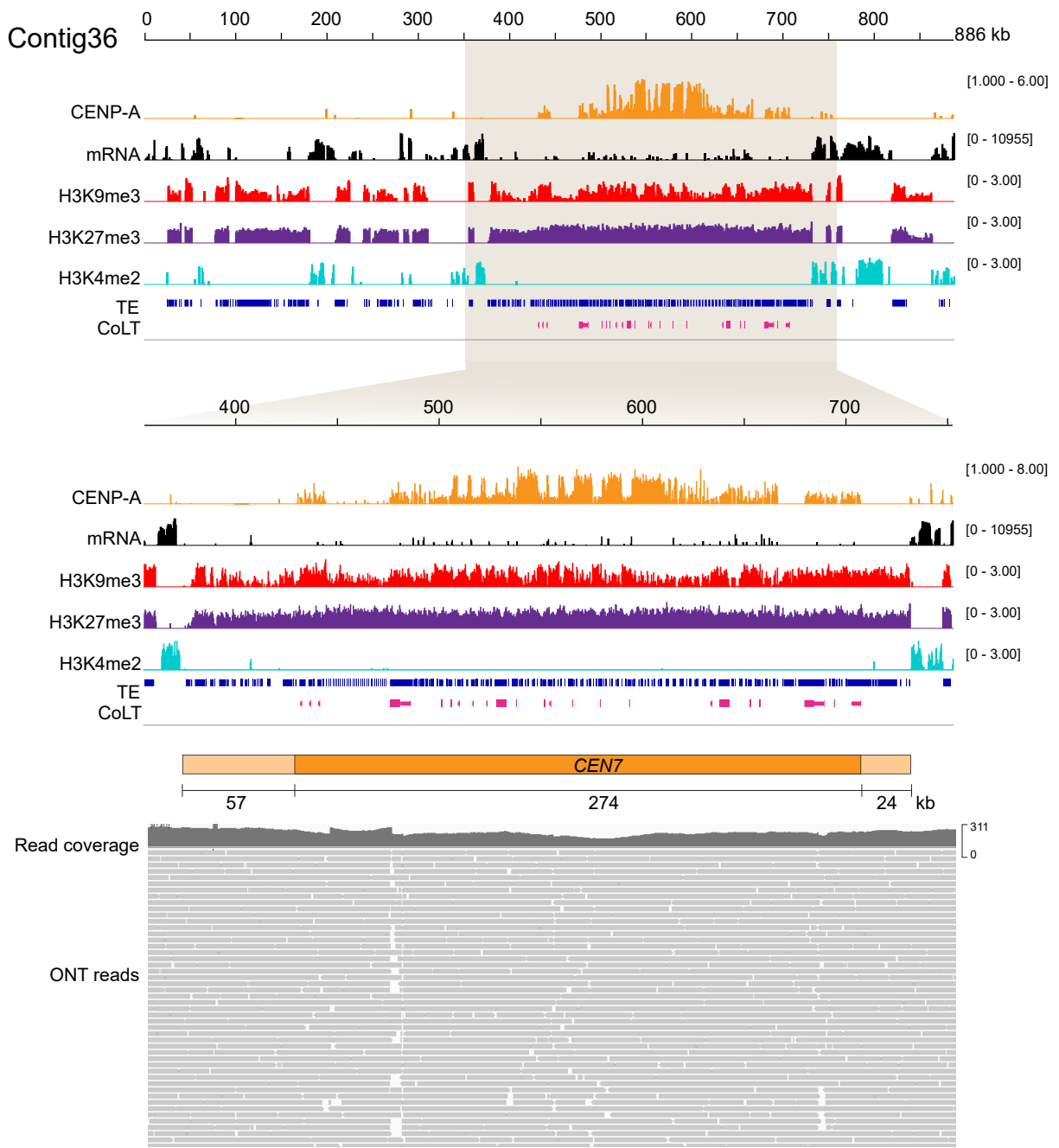

H

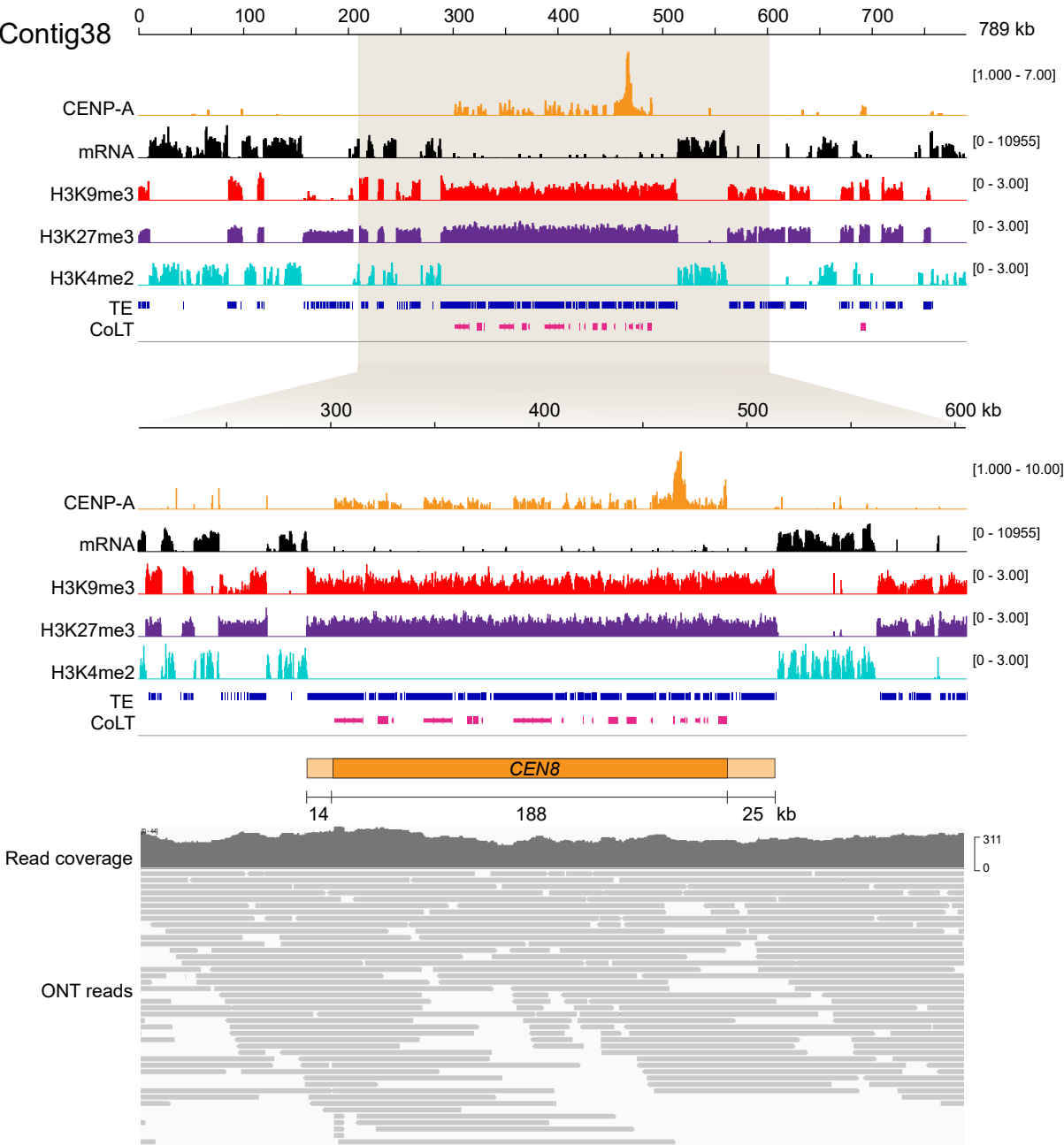

I

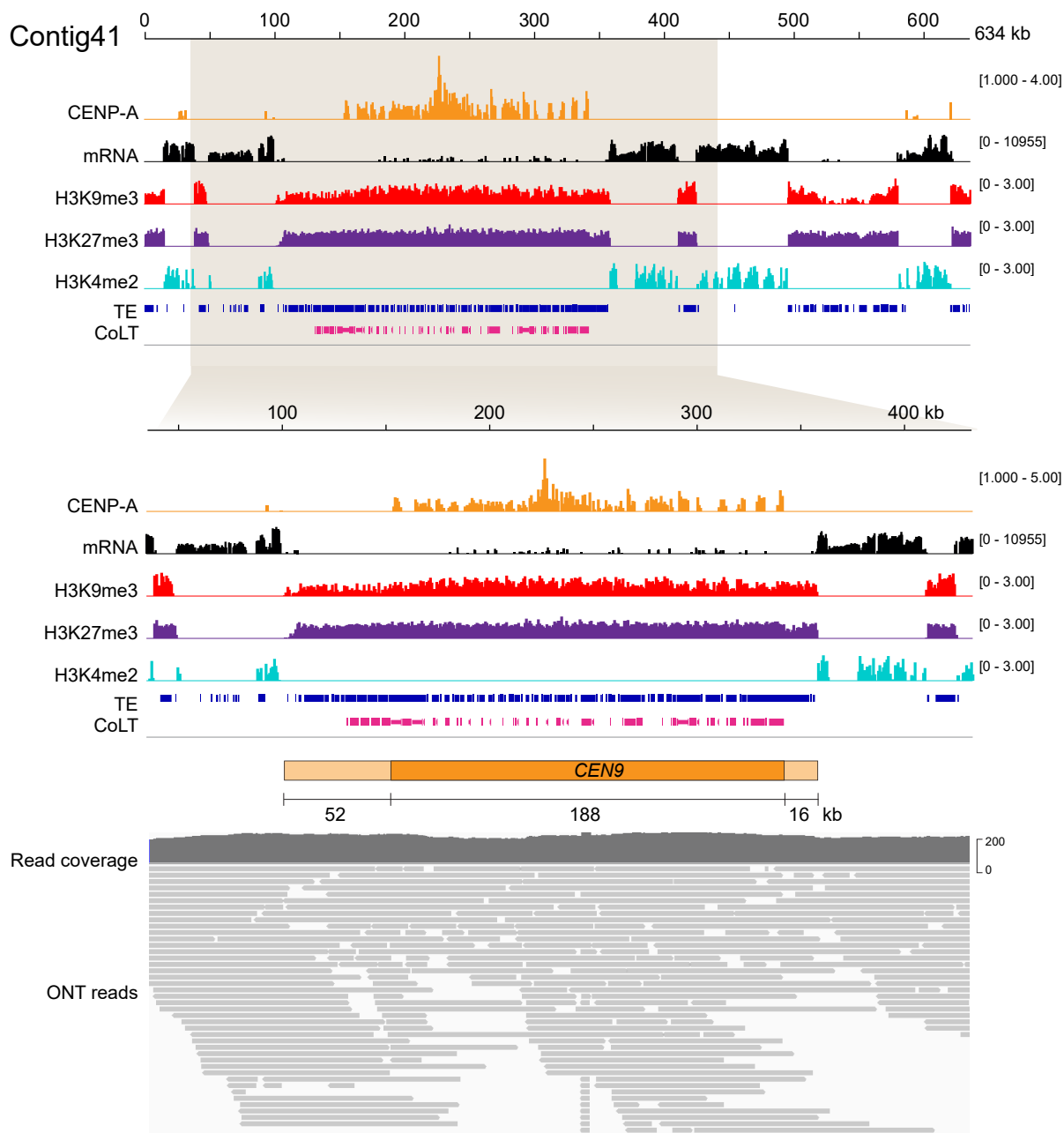

J

Contig51

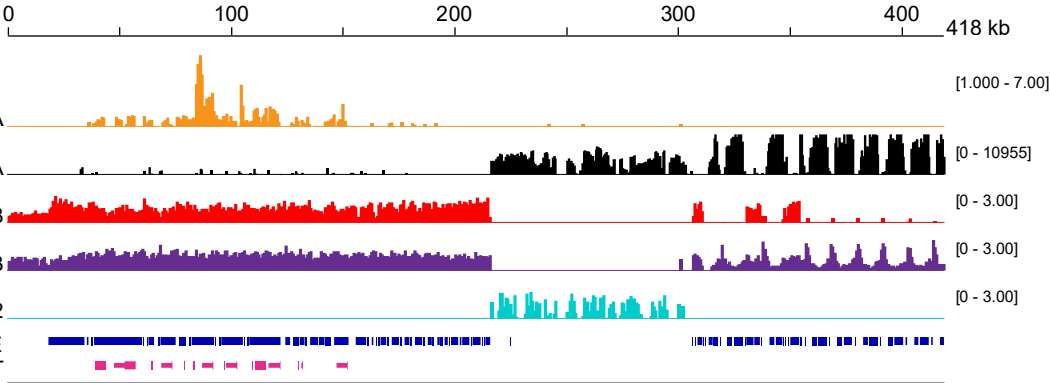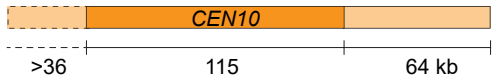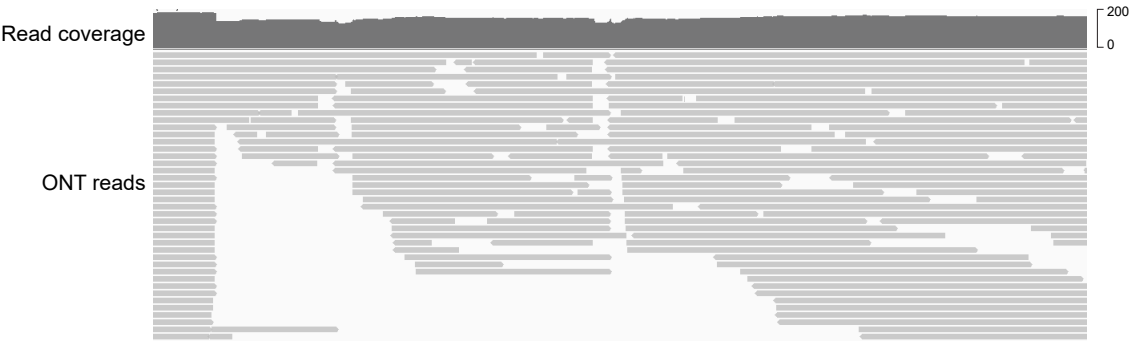

Supplement: S7 Fig — For each panel (A-J), upper, an overview of the contig exhibiting enrichments of both CENP-A and histone modifications (H3K9me3, H3K27me3 and H3K4me2), and distribution of transposable elements. TE, transposable elements. CoLT, Copia-like transposon. Middle, a magnified view of the region shaded in the contig shown above; a diagram displaying the correlation of core centromere (CENP-A binding region) and heterochromatic regions. A 400 kb region is shown for each centromere that has assembled pericentromeric regions on both sides. A 270 kb region is shown for CEN5, as it is missing one side of the intact pericentric region. The length of Contig 51 is close to 400 kb, only the entire contig is shown (J). Bottom, an image showing long-read coverage. Canu-corrected Nanopore reads were mapped to all centromeres using Minimap2 [90], except CEN7, which was verified by all Nanopore and PacBio reads (without Canu-correction) using GraphMap [91]. For better visualization, indels of reads were masked. Asterisk in (B), regions (underlined) that have high CENP-A enrichment but show no CoLT and very low TE density contains unknown repetitive sequences unique to CEN2. (PDF) [file pgen.1008646.s007.pdf]

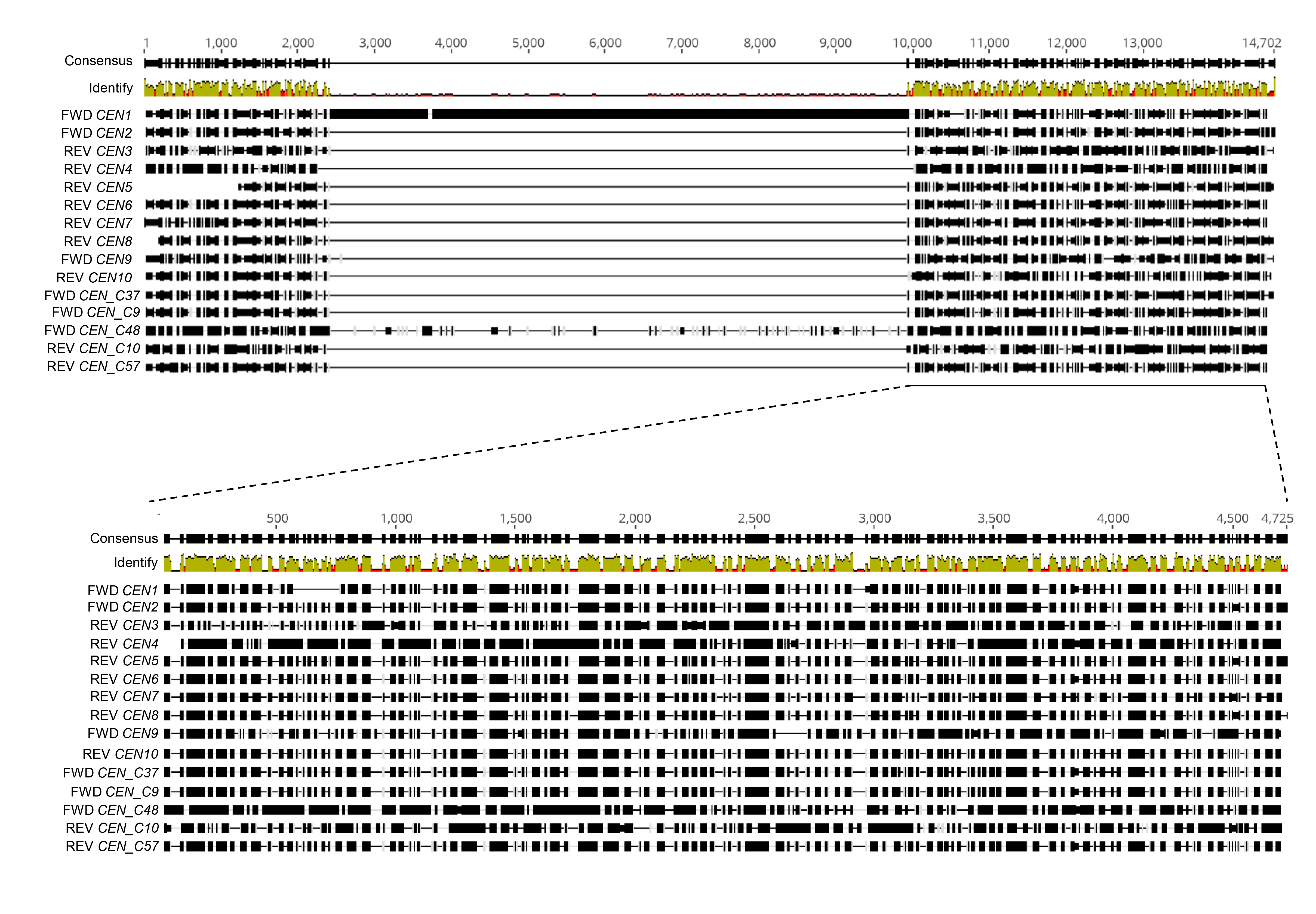

Supplement: S8 Fig — Upper panel, alignment of CENP-A binding regions. Lower panel, magnified view of the conserved 5 kb region. Images were adapted from the alignment result generated by Geneious. (TIF) [file pgen.1008646.s008.tif]

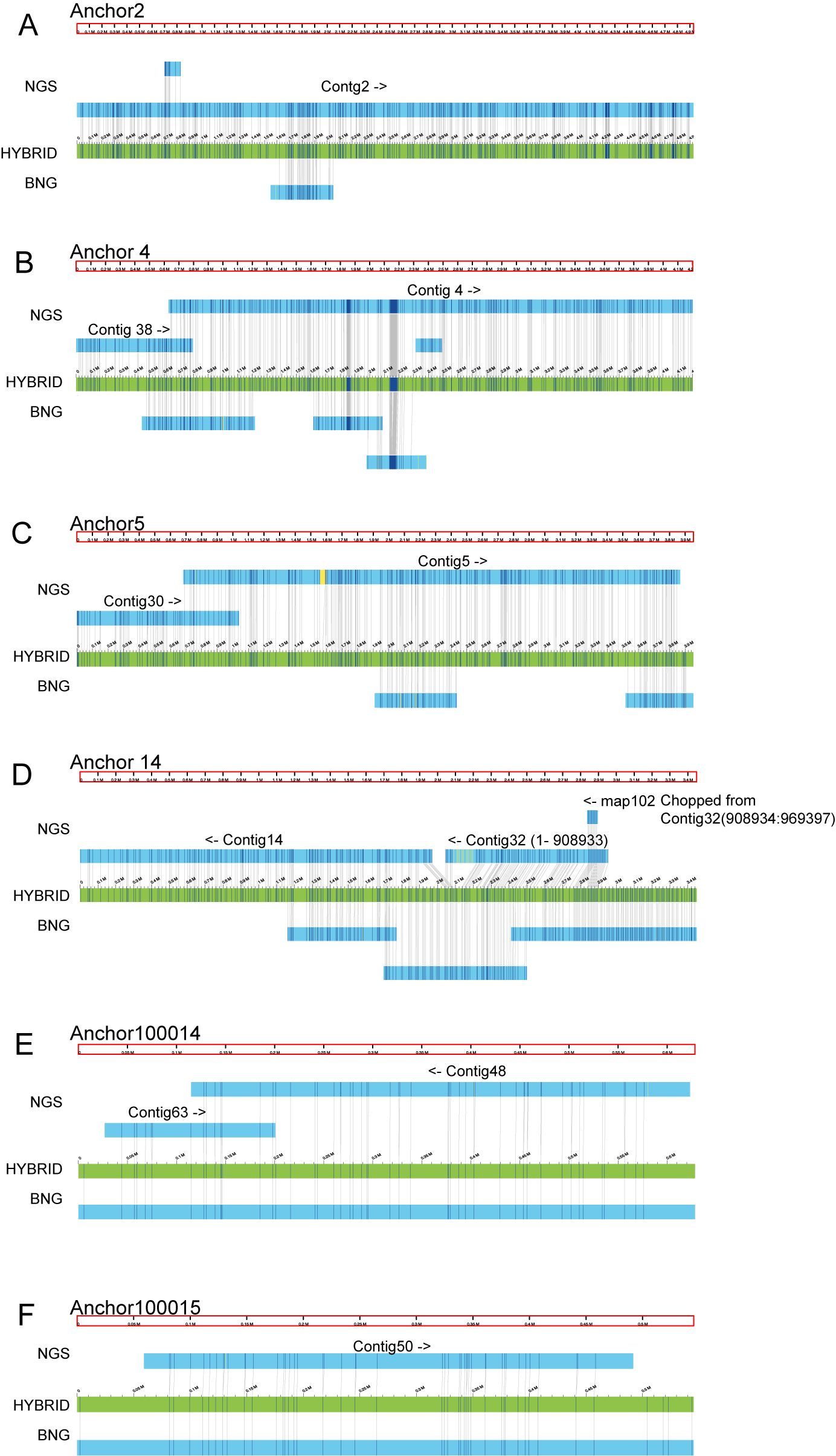

Supplement: S9 Fig — (A) Contig 2 representing most of the cases that only parts of contigs were anchored by Bionano molecules. (B) One case indicating that two contigs (Contig 5 and Contig 30) were not anchored by Bionano but suggested to be combined. (C-E) Three cases displaying that contigs were anchored by Bionano and suggested to be combined. (E, F) Two cases showing that contigs can be fully covered by Bionano molecules. Map102 is a fragment trimmed from Contig 32. NGS, next generation sequencing, namely Psojae2019.1; BNG, Bionano de novo assembly; HYBRID, hybrid scaffold. (TIF) [file pgen.1008646.s009.tif]

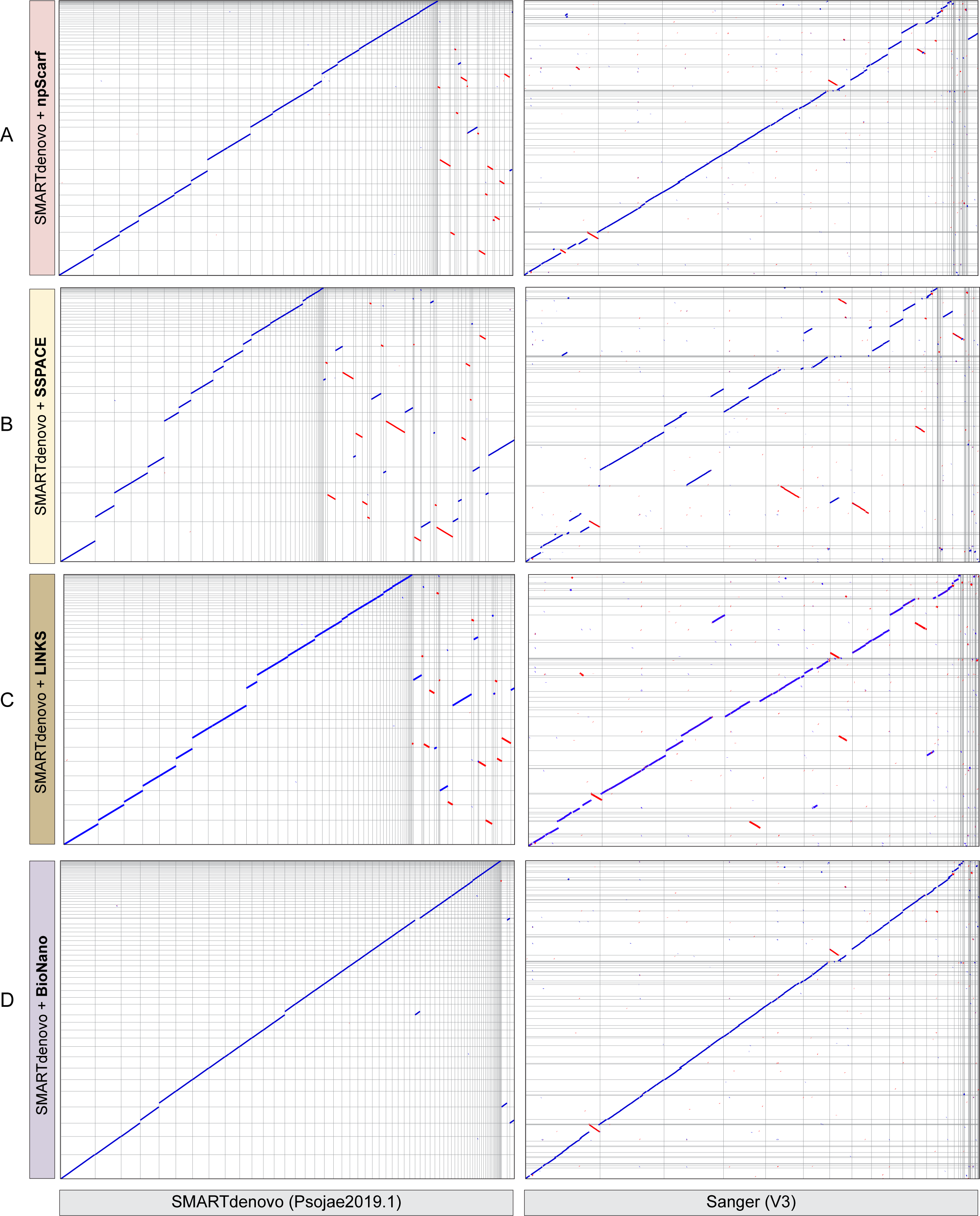

Supplement: S10 Fig — (A-D) Assemblies generated by the scaffolding programs npScarf, SSPACE and LINKS, and Bionano mapping were aligned respectively to the original SMARTdenovo assembly (Psojae2019.1), as well as the Sanger genome, and plotted utilizing the MUMmer package [92]. (TIF) [file pgen.1008646.s010.tif]
