# Supplementary material for: Long transposon-rich centromeres in an oomycete reveal divergence of centromere features in Stramenopila-Alveolata-Rhizaria lineages
Source: PLoS Genet. 2020 Mar 9;16(3):e1008646. doi: 10.1371/journal.pgen.1008646 (PMC7082073; doi:10.1371/journal.pgen.1008646)
Supplement: S6 Fig — In all panels (A-H), the outer tracks (track A) illustrate assembled contigs (in Psojae2019.1) or scaffolds (in P. sojae V3), and are color coded as given in key at the top. Yellow regions on the outer tracks indicate the location of centromeres (CENP-A binding regions). Tracks B-E show the location of other genomic features as given in the key on the bottom. Blue and orange lines in track F link regions with synteny extending over 2 kb, with orange lines corresponding to inversions. To demonstrate sequence gaps in the Sanger assembly more clearly, black dots representing assembly gaps are also shown between track E and F. Names of contigs that contain P. sojae centromeres are enclosed in circles. Arrowheads indicate the shrunk centromeres present in the Sanger scaffolds. (A) Comparison of Sanger Scaffold 2 (sca2) and its syntenic contigs in the Psojae2019.1 assembly. (B) Comparison of Sanger Scaffold 8 (sca8) and its syntenic contigs in the Psojae2019.1 assembly. (C) Comparison of Sanger Scaffold 9 (sca9) and Scaffold 12 (sca12), and their syntenic contigs in the Psojae2019.1 assembly. (D) Comparison of Sanger Scaffold 4 (sca4) and its syntenic contigs in the Psojae2019.1 assembly. Dots under CEN4 indicate the regions showing CENP-A peaks, as a transcriptionally active region is found in CEN4. (E) Comparison of Sanger Scaffold 3 (sca3) and its syntenic contigs in the Psojae2019.1 assembly. (F) Comparison of Sanger Scaffold 6 (sca6) and its syntenic contigs in the Psojae2019.1 assembly. Dashed lines under Psojae2019.1 Contig 20 and Contig 34 indicate duplicated regions. (G) Comparison of Sanger Scaffold 5 (sca5) and its syntenic contigs in the Psojae2019.1 assembly. (H) Comparison of Sanger Scaffold 1 (sca1) and its syntenic contigs in the Psojae2019.1 assembly. The two centromeric regions in Scaffold 1 of the Sanger assembly (P. sojae V3) suggested by CENP-A ChIP-seq are indicated by arrows. Part of Contig 4 is inverted in the Psojae2019.1 assembly compared to the [file pgen.1008646.s006.pdf]

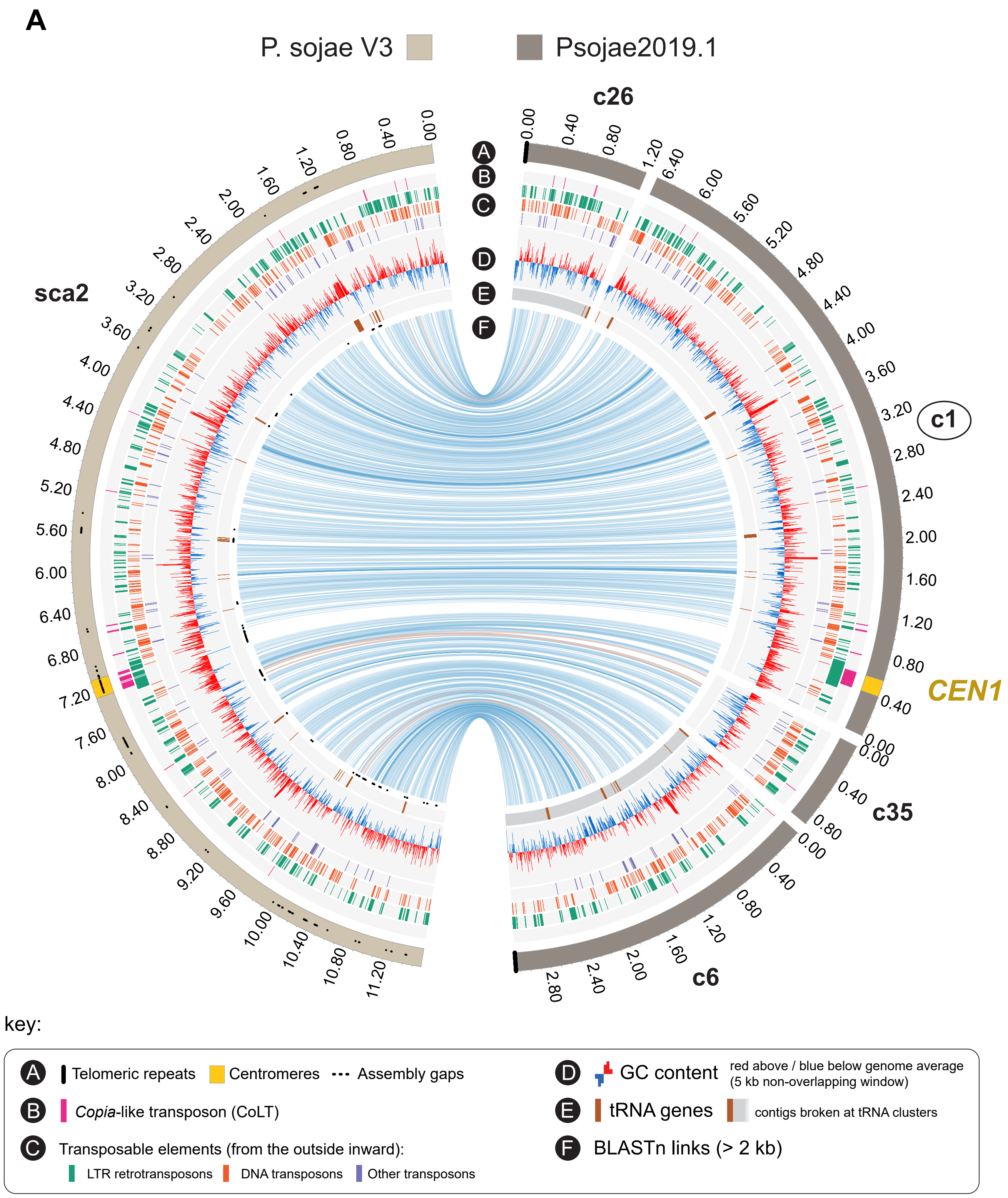
A
P. sojae V3
Psojae2019.1
c26
c1
c35
c6
sca2
CEN1
A
B
C
D
E
F
key:
Telomeric repeats
Centromeres
Assembly gaps
Copia-like transposon (CoLT)
Transposable elements (from the outside inward):
LTR retrotransposons
DNA transposons
Other transposons
GC content
red above / blue below genome average
(5 kb non-overlapping window)
tRNA genes
contigs broken at tRNA clusters
BLASTn links (> 2 kb)

B

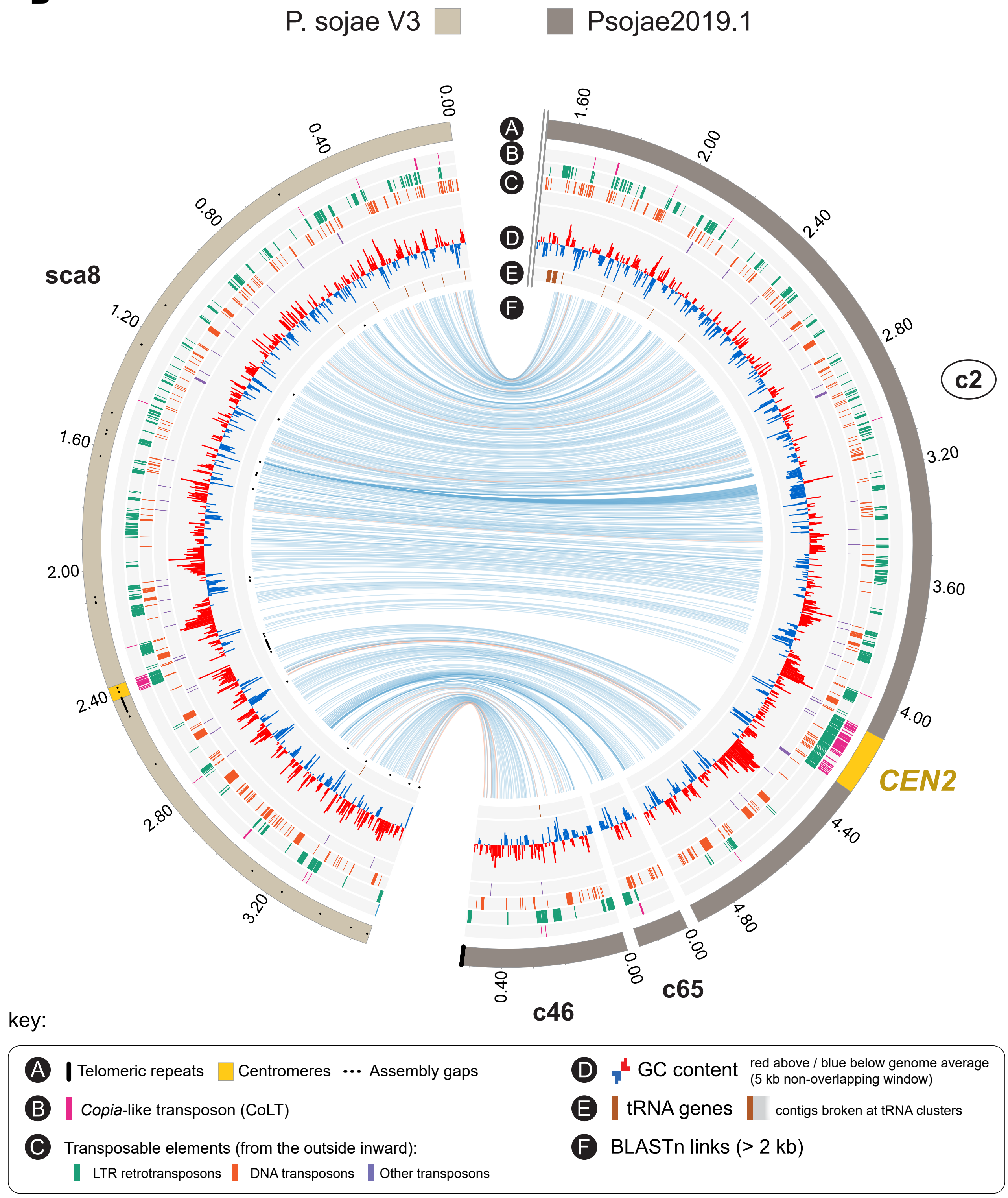
P. sojae V3
Psojae2019.1
sca8
c2
CEN2
c46
c65
A
B
C
D
E
F
0.00
0.40
0.80
1.20
1.60
2.00
2.40
2.80
3.20
1.60
2.00
2.40
2.80
3.20
3.60
4.00
4.40
4.80
0.00
0.00
0.40
key:
A Telomeric repeats Centromeres Assembly gaps
B Copia-like transposon (CoLT)
C Transposable elements (from the outside inward):
LTR retrotransposons DNA transposons Other transposons
D GC content red above / blue below genome average (5 kb non-overlapping window)
E tRNA genes contigs broken at tRNA clusters
F BLASTn links (> 2 kb)

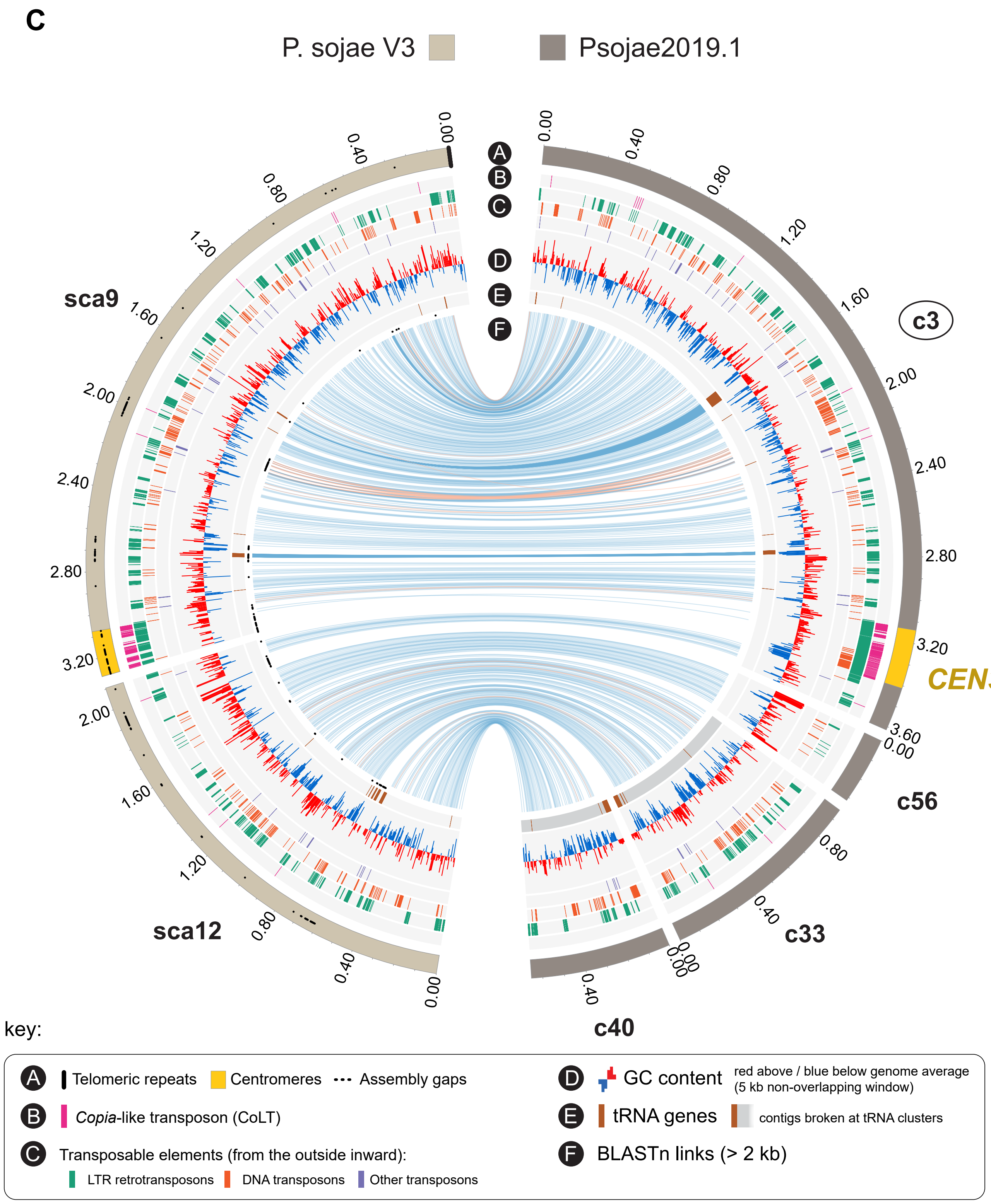
C
P. sojae V3
Psojae2019.1
A
B
C
D
E
F
sca9
sca12
c3
c56
c33
c40
CEN3
key:
A Telomeric repeats Centromeres Assembly gaps
B Copia-like transposon (CoLT)
C Transposable elements (from the outside inward):
LTR retrotransposons DNA transposons Other transposons
D GC content red above / blue below genome average (5 kb non-overlapping window)
E tRNA genes contigs broken at tRNA clusters
F BLASTn links (> 2 kb)

D

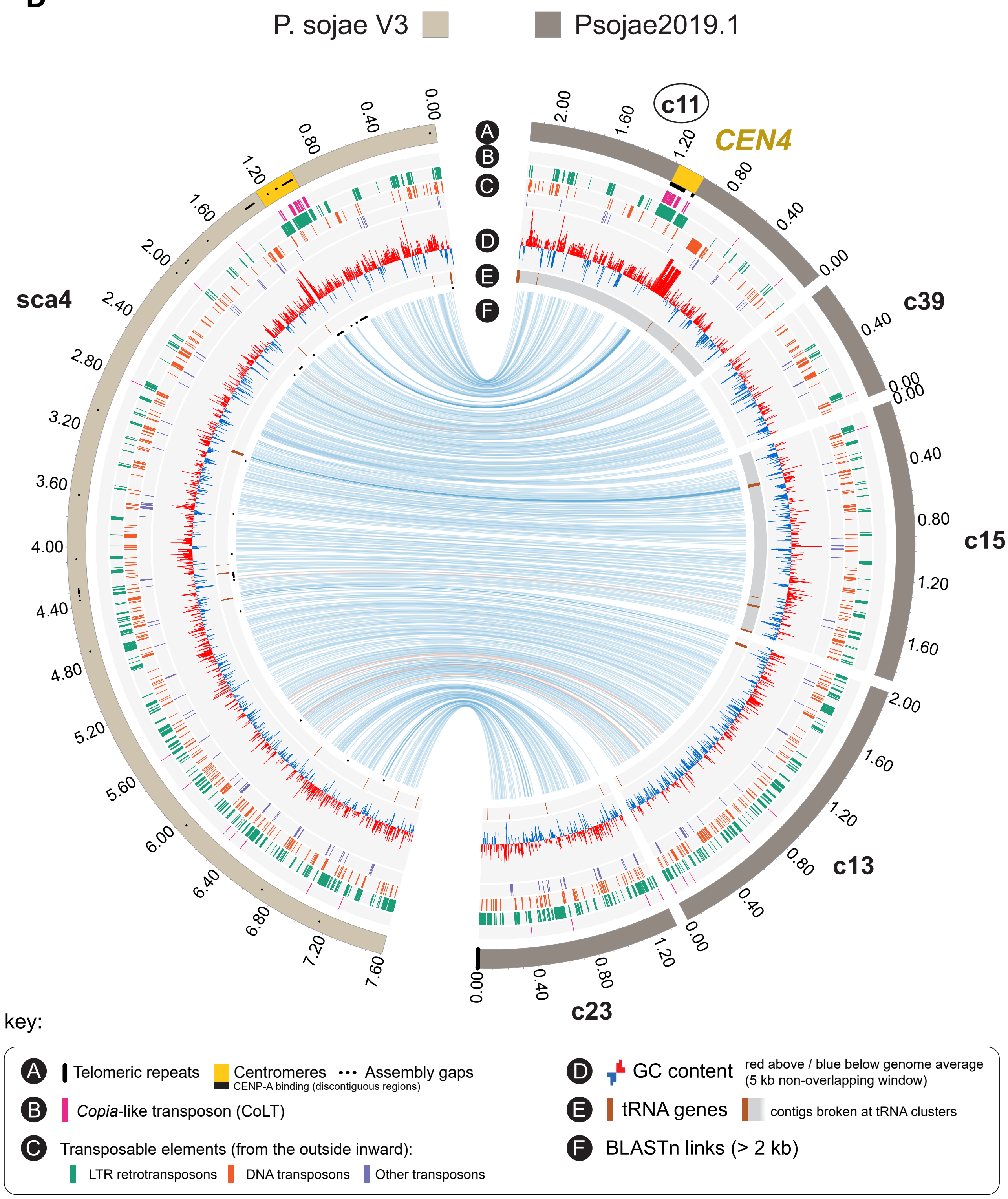
P. sojae V3
Psojae2019.1
c11
CEN4
sca4
c39
c15
c13
c23
A
B
C
D
E
F
key:
A Telomeric repeats
Centromeres
CENP-A binding (discontiguous regions)
Assembly gaps
B *Copia*-like transposon (CoLT)
C Transposable elements (from the outside inward):
LTR retrotransposons
DNA transposons
Other transposons
D GC content
red above / blue below genome average
(5 kb non-overlapping window)
E tRNA genes
contigs broken at tRNA clusters
F BLASTn links (> 2 kb)

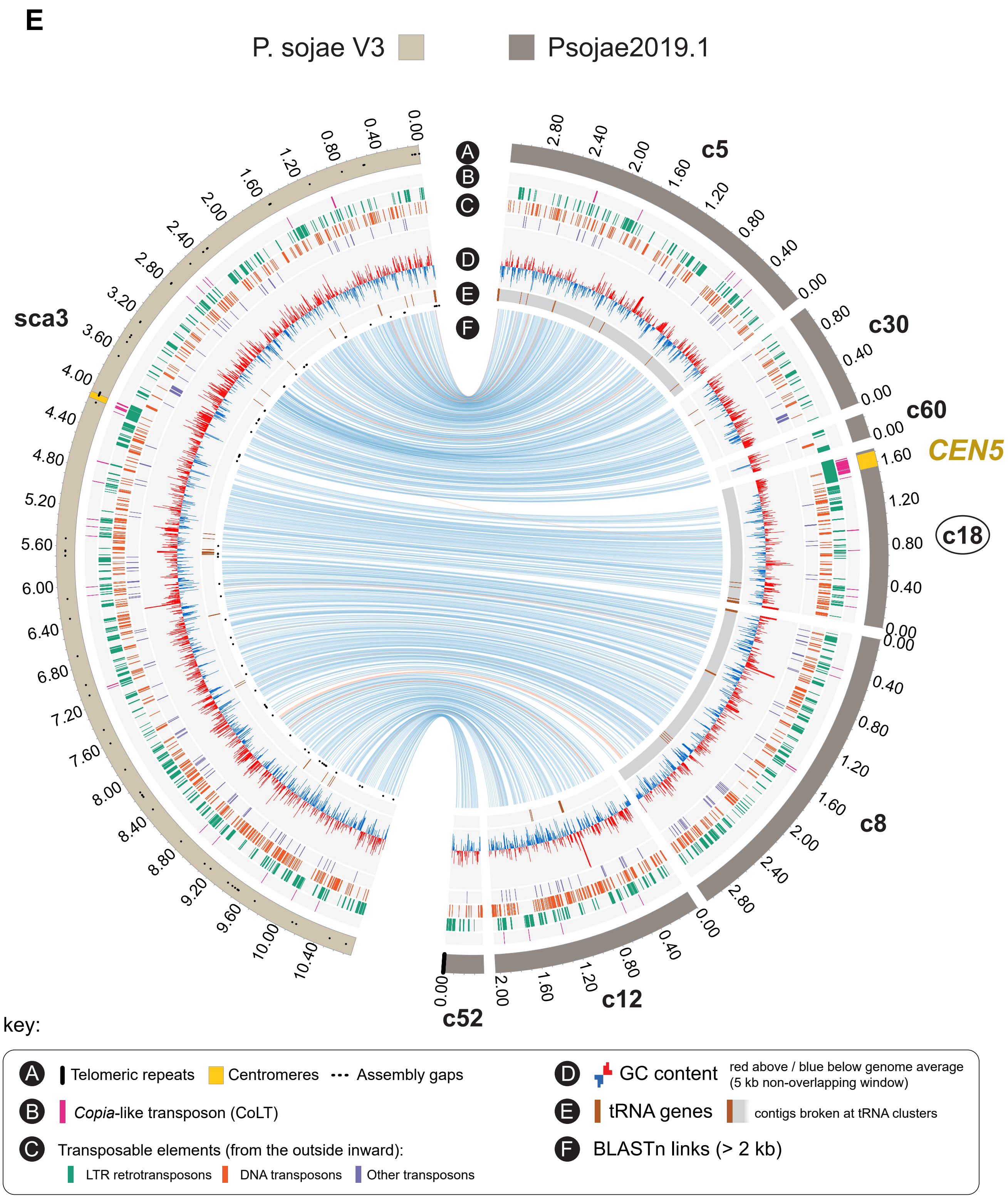
E
P. sojae V3
Psojae2019.1
A
B
C
D
E
F
sca3
c5
c30
c60
CEN5
c18
c8
c12
c52
key:
A Telomeric repeats
Centromeres
Assembly gaps
B Copia-like transposon (CoLT)
C Transposable elements (from the outside inward):
LTR retrotransposons
DNA transposons
Other transposons
D GC content
red above / blue below genome average (5 kb non-overlapping window)
E tRNA genes
contigs broken at tRNA clusters
F BLASTn links (> 2 kb)

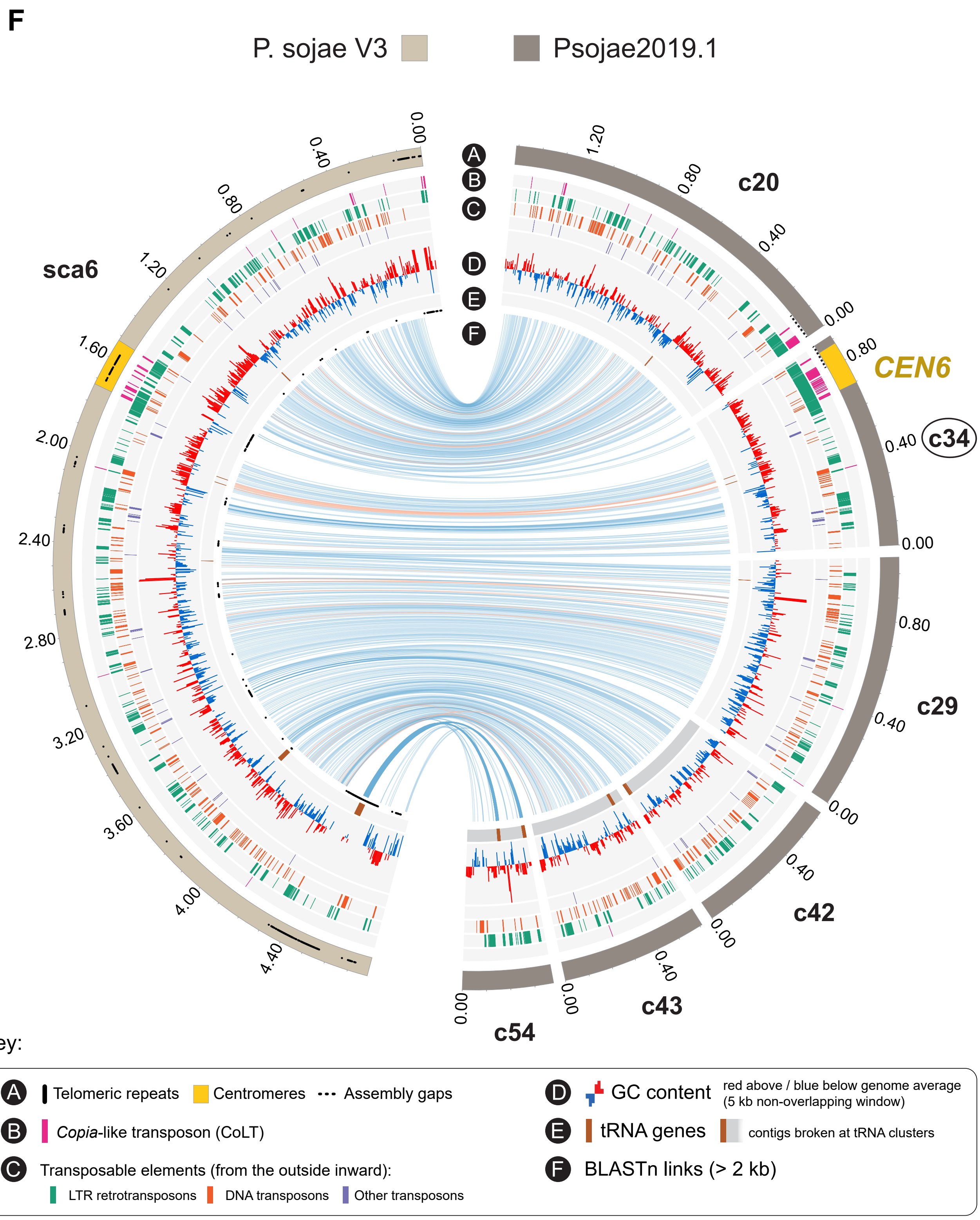

F
P. sojae V3
Psojae2019.1
sca6
c20
c34
CEN6
c29
c42
c43
c54
A
B
C
D
E
F
key:
A Telomeric repeats
Centromeres
Assembly gaps
B Copia-like transposon (CoLT)
C Transposable elements (from the outside inward):
LTR retrotransposons
DNA transposons
Other transposons
D GC content
red above / blue below genome average
(5 kb non-overlapping window)
E tRNA genes
contigs broken at tRNA clusters
F BLASTn links (> 2 kb)

G

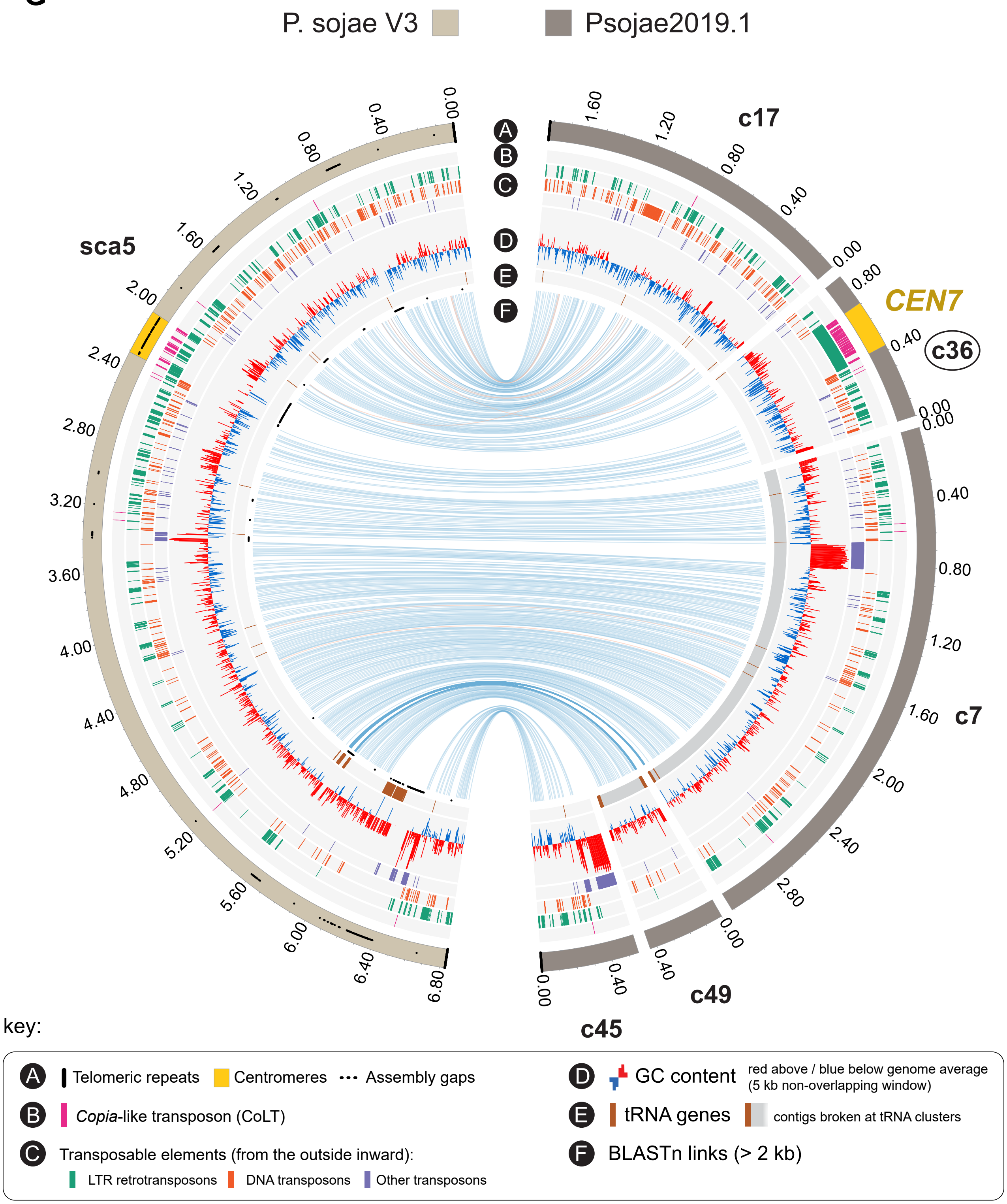
P. sojae V3
Psojae2019.1
sca5
c17
c36
CEN7
c7
c49
c45
A
B
C
D
E
F
key:
A Telomeric repeats
Centromeres
Assembly gaps
B *Copia*-like transposon (CoLT)
C Transposable elements (from the outside inward):
LTR retrotransposons
DNA transposons
Other transposons
D GC content
red above / blue below genome average
(5 kb non-overlapping window)
E tRNA genes
contigs broken at tRNA clusters
F BLASTn links (> 2 kb)

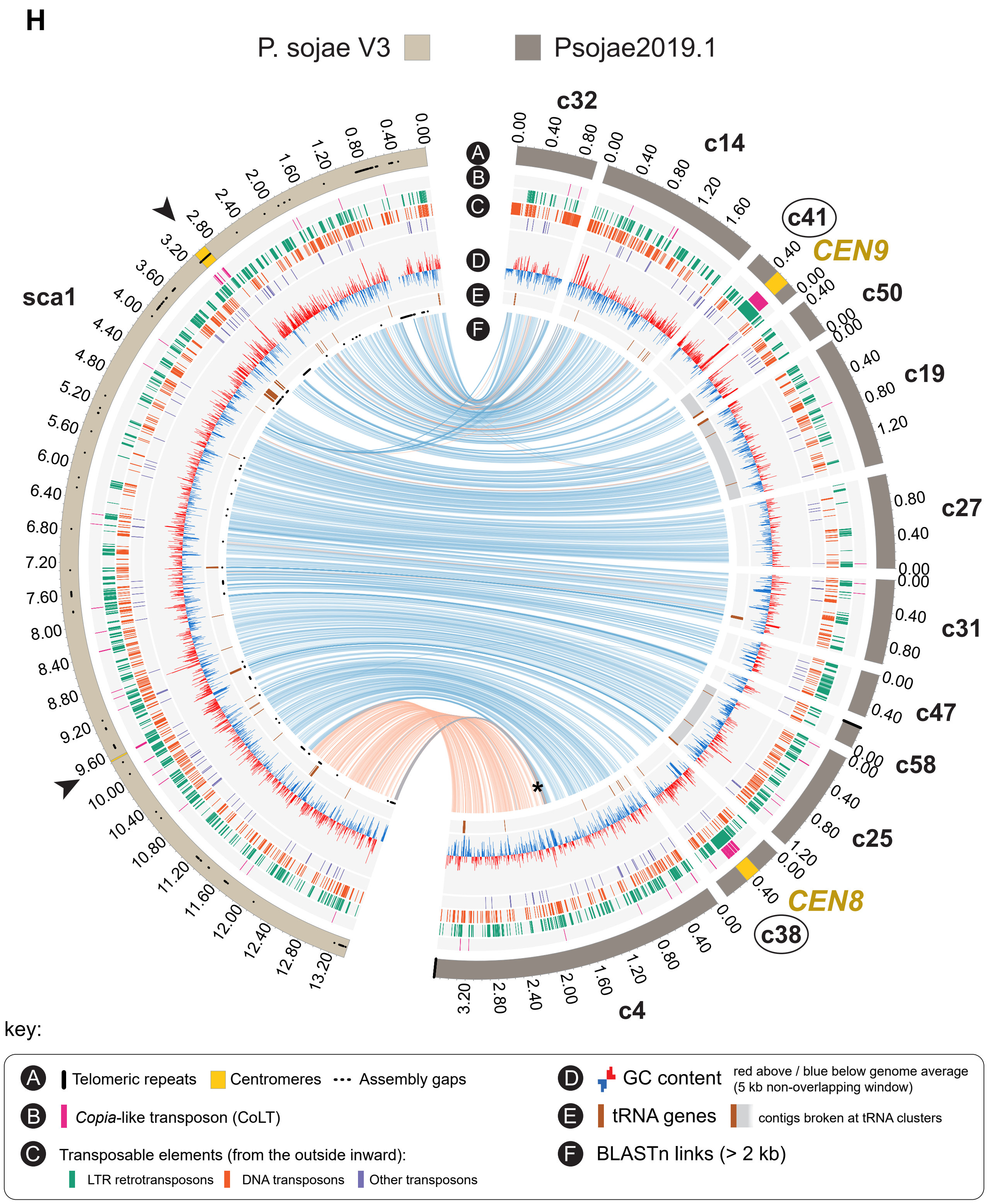
H
P. sojae V3
Psojae2019.1
sca1
c32
c14
c41
CEN9
c50
c19
c27
c31
c47
c58
c25
CEN8
c38
c4
A
B
C
D
E
F
*
key:
A Telomeric repeats
Centromeres
Assembly gaps
B Copia-like transposon (CoLT)
C Transposable elements (from the outside inward):
LTR retrotransposons
DNA transposons
Other transposons
D GC content
red above / blue below genome average
(5 kb non-overlapping window)
E tRNA genes
contigs broken at tRNA clusters
F BLASTn links (> 2 kb)
