# Supplementary material for: Long transposon-rich centromeres in an oomycete reveal divergence of centromere features in Stramenopila-Alveolata-Rhizaria lineages
Source: PLoS Genet. 2020 Mar 9;16(3):e1008646. doi: 10.1371/journal.pgen.1008646 (PMC7082073; doi:10.1371/journal.pgen.1008646)
Supplement: S2 Table — (DOCX) [file pgen.1008646.s012.docx]

**S2 Table. Statistics of ChIP-seq samples.**

| Sample name | Accession no. (NCBI) | Total reads | Reads mapped | % mapped |
| --- | --- | --- | --- | --- |
| GFP-CENP-A_ Input | [SRR10080681](https://dataview.ncbi.nlm.nih.gov/object/SRR10080681) | 45,803,908 |  | 84.48 |
| GFP-CENP-A_IP | SRR10080680 | 40,301,524 |  | 81.35 |
| H3K27me3_Input | SRR10080681 | 45,803,908 |  | 84.48 |
| H3K27me3_IP | SRR10080679 | 30,980,430 |  | 80.45 |
| H3K9me3_Input | SRR10206162 | 33,032,101 | 32,234,974 | 97.59 |
| H3K9me3_IP | SRR10828930 | 33,510,407 | 33,053,755 | 98.64 |
| H3K4me2_Input | SRR8633151 | 34,292,628 | 33,656,800 | 98.15 |
| H3K4me2_IP | SRR10828931 | 30,278,995 | 29,666,330 | 97.98 |
