# Supplementary material for: Long transposon-rich centromeres in an oomycete reveal divergence of centromere features in Stramenopila-Alveolata-Rhizaria lineages
Source: PLoS Genet. 2020 Mar 9;16(3):e1008646. doi: 10.1371/journal.pgen.1008646 (PMC7082073; doi:10.1371/journal.pgen.1008646)
Supplement: S4 Table — (DOCX) [file pgen.1008646.s014.docx]

**S4 Table. Five incompletely assembled centromeres in the Psojae2019.1 assembly and their corresponding CENP-A regions mapped in the Sanger assembly.**

| Psojae2019.1 | | | | |  | | Sanger V3 | | |
| --- | --- | --- | --- | --- | --- | --- | --- | --- | --- |
| Name | Contig | Position of mapped CENP-A region (kb) | GC% of *CEN* |  | | Scaffold | | Position of *CEN* (kb) |  |
| *CEN_C9** | 9 | 1-158 | 50.76 |  | | 13 | | 1690-1879 |  |
| *CEN_C48** | 48 | 1-35 | 45.25 |  | |  |  |  |  |
| *CEN_C10* | 10 | 2248-2312 | 57.23 |  | | 11 | | 118 -143 |  |
| *CEN_C37* | 37 | 721-771 | 56.26 |  | | 21 | | 61-103 |  |
| *CEN_C57* | 57 | 229-273 | 56.51 |  | | 14 | | 1716-1806† |  |

**CEN_C9* and *CEN_C48*, sequences surrounding the two incompletely assembled centromeres are collinear with Sanger Scaffold13, indicating that *CEN_C9* and *CEN_C48* may be parts of the same centromere.

†The disrupted centromere in Sanger Scaffold 14 was identified by synteny analysis. The start of the centromere position (i.e. 1716 kb) is a transcriptional region that is syntenic to the one next to *CEN_C57*.
