# Supplementary material for: Long transposon-rich centromeres in an oomycete reveal divergence of centromere features in Stramenopila-Alveolata-Rhizaria lineages
Source: PLoS Genet. 2020 Mar 9;16(3):e1008646. doi: 10.1371/journal.pgen.1008646 (PMC7082073; doi:10.1371/journal.pgen.1008646)
Supplement: S5 Table — (DOCX) [file pgen.1008646.s015.docx]

**S5 Table. *P. sojae* strains used in the study.**

| Strain Name | Genotype | Source |
| --- | --- | --- |
| P6497 | Wild type |  |
| YFP09 | *P. sojae* P6497::pYF3-GFP-CENP-A | This study |
| YFP10a1 | *P. sojae* P6497 *cenpa*Δ::*GFP-CENP-A* | This study |
| YFP10b1 | *P. sojae* P6497 *cenpa*Δ::*GFP-CENP-A* | This study |
