# Supplementary material for: Long transposon-rich centromeres in an oomycete reveal divergence of centromere features in Stramenopila-Alveolata-Rhizaria lineages
Source: PLoS Genet. 2020 Mar 9;16(3):e1008646. doi: 10.1371/journal.pgen.1008646 (PMC7082073; doi:10.1371/journal.pgen.1008646)
Supplement: S6 Table — (DOCX) [file pgen.1008646.s016.docx]

**S6 Table. Primers used in this study.**

| Name | Sequence | Usage |
| --- | --- | --- |
| JOHE45057 | CACAGCTCCCAGACGCAAGC | 3' RACE of CENP-A |
| JOHE45486 | ACACTGGCGGCCGTTACTAGTGCGCTGTCCAAGCGCGCG | Clone GFP-CENP-A HDR template using NEBuilder® HiFi DNA Assembly |
| JOHE45487 | CCTTGCCCATCGCTGCCTGCCTGACTGC |  |
| JOHE45488 | GCAGGCAGCGATGGGCAAGGGCGAGGAA |  |
| JOHE45489 | GCGATGCCATCTTGTAGAGTTCATCCATGCCATGC |  |
| JOHE45490 | ACTCTACAAGATGGCATCGCCGCGTCCA |  |
| JOHE45491 | GACCATGATTACGCCAAGCTTGCGCTATACTACGCCGACCA |  |
| JOHE50156 | CTAGCACTGCTCTGATGAGTCCGTGAGGACGAAACGAGTAAGCTCGTCAGCAGTCAGGCAGGCAGCGA | sgRNA_PsCENPA_182 forward |
| JOHE50157 | AAACTCGCTGCCTGCCTGACTGCTGACGAGCTTACTCGTTTCGTCCTCACGGACTCATCAGAGCAGTG | sgRNA_PsCENPA_182 reverse |
| JOHE50062 | 5' GTTCATTTAGGGAGGTGCCACTGTA 3' | CENP-A_Junction_5' |
| JOHE50063 | 5' TCCTGCTGAAGGGGAAATAGATG 3' | CENP-A_Junction_3' |
| JOHE45358 | CGTTCACATCACCATCCAGTTCCAC | GFP_Seq_R |
| JOHE45420 | GACGGCTGCTGGGATCACGC | GFP_Seq_F |
