# Supplementary material for: Long transposon-rich centromeres in an oomycete reveal divergence of centromere features in Stramenopila-Alveolata-Rhizaria lineages
Source: PLoS Genet. 2020 Mar 9;16(3):e1008646. doi: 10.1371/journal.pgen.1008646 (PMC7082073; doi:10.1371/journal.pgen.1008646)
Supplement: S3 File — (DOCX) [file pgen.1008646.s021.docx]

**S3 File.** **The** **5 kb consensus sequence shared by *P. sojae* centromeres, and representative full-length CoLT homologs found in the *P. sojae*, *B. lactucae* and *P. citricola* genome assemblies.**

>5kb_common_sequence_consensus

CGGATCGGAGCGTGATGGAAGGTATGGATCTTGGACACGAAGCTCGGACGAAGCGGAGGGAGGGACGTCGCAGACGGCGGGACCGGAGCGAAGGCACTTGAGGGGTAGAATGAAGATTCGAAAGTGATTGAATTGTTGGAGCTTACACCCTACTGTTATAGACACGCGACCCTGCGTCCTATCACCTAGTGCATCTAATCAACCATCACTCACCGGTGTTCATCCAATCATCCTACTCGTCTAATCATCGGTGGCGTAAACAACTTACCGTAATCACCATAATCAACGTGGGTTCGGATTGGACCGGTTCGGGTTAACCGGTTAATGGGTACAACTGGTACACAGTACCCAAGACCAGCAACCCTCAATACGCCCCCTCAATGCGAAGTCCCACGAATGCCGAGAGCCACCGTCAGATCCGTGTGGCGTGGTCCCGCAAGCGCCTTCGTCATGATGTCCGCTAGCATCACAGAAGTCTCGCAGTACTGGAGCTTGACTTCGCCGCGCTTGACTTCATCGCGGATGTGGTGGTACTTGATGTCGATGTGCTTGGCGCGACCGTGGTTCACTGGGTTCTTCGTCATCTTGATGCACGACTGGTTGTCTTCGCGGATCATGAGTTCGGGTCCAGTCTCATTAGCGGCCGCCAGAATCTCGCATAGCAGGCGATGAATCCACTTGCCTTCTTGGATAGCCAGACTGAGCGCGATGTACTCCGCTTCGCTCGTGGACAGCGACACACTTGATTGCTTCTTGCTTCCCCAGCTCACAGGCGCACTCATGAGCATGAACGTGTAGCCTGAAGTGGACTTGCGGTCGGCGAGGTCGCCGGCCCAGTCCGCGTCCGAGTAGCCACGAAAGTCGATCTTGTCGCCCGGCTTGAAGCAGATCCCATGCGTCTTGGTGCCTTGCAGGTAGCGAAAGATACGCTTGACCGCAACCCAGTGTTCCTCTTGGGGGTTCTCCATGAAGCGCGATACATAGCCAACGGCGTACGCGATGTCTGGGCGCGTGGCGGTCATCAGGTGCATCAGAGCACCAACAGCTTCGCGAAACGGAGCGTTCACCTTGGTCGCCGCGTCGCTTGGAACCAGTCGAGTGCTCATGTCCACCGGACTCACCACGGCCTTGCACTCGTCCATGGCGAAGCGCTTCAGGATGTCGTCCACGTATCGCCGCTGGCACATGGTCACGCTGCCGTCCGGGCCGTCCACCAACTCGATCCCAAGAACAAACGCGCACTTGCCGCTGTCGGTCATCTCGAAGCGCGTCTTCAGGTCGGTCTTCGTGCGTGCGATCAGCTCGGGCGAACTCCCGGTAATCAGTACGTCATCCACGTACACCAGTACAAGCACGCAGTGACCGTCCACCACCTTGATGTAGAGGCACGGATCGAAGGCCGACACTTGGAAGCCGATGGAGCACACGAACTCGTCGAAGGTCTCGTTCCACACTCGCGAAGCTTGCTTGAGACCGTAGATGGCCTTCACCAGTTCCAGGCAGTCGAAGTCACCATCCAGTTCGACGCCTTCCGGAACCACGCAGAAGACCACCTCCTTCATTATCCCGTACAGGAATGCTGTCACCACGTCGAGTTGGTCGAGGGGCCAGCCAAAATACTTAGCAATCGCGATCACCATGCGCAGCGTCACATACTTGACCACGGGCGAGAACGTCTCGGTGTAGTCAATGCCGTACTTCTGCTTGAAGCCCTTGGCCACGAGGCGCGCCTTGTACTTCTCGATCGAGCCGTCCGCTTTGCGCTTGATCTTGAACACCCACTTGGTATCGATGGCGCGTTGTCCGTTAGGCAACTTGGCAGCTCGAAACACACCACGCAGTCGCATCGACTTGAGCTCCGCGCGGATCGCTTTGCGCCAGTGCACTTGATCAGGACCGTTCACAGCCTCTTGGAACGTCGTCGGCTCCGATAGATCGGCTGCTGCTTCCACAGCGTTGGCACTGGCACGCCAAAACACTGGAGGCGTCGAGTCGTCGTCTTCATCTTGGTCCCCAGACTTTTCTTCTTCGTCGGGGTCGGCTCGGCGCGAGGAGGATACGTCCGGCGCACTTGCTTCTTCTAAGCCAGCTCGGTGGCGAACGGGGCGCGATCTTCTGGCAGTGTTGTCGTCGCCACTCGGTCGGTACTTGCGCTTCCCCGCCTGCTTGAAGCTCGTTTGGCGCGGTCCATCGTTGTCGTCCAGGTCCAGCGCGTCCAAGTCCAGCACAGCATCATCGACGTCTTCATCCGAAGTATGCGTCGAAAGTCCAAAGGTCGACTCATCGAAGTTGACATCGCGACTGATAACCACCTGCCCCGCCTCGATGTCATAAACTCGATACGCCTTGGACACTTCTTCGTAGCCGAGGAACAGCCCGGCGCGAGCCTTGGGGTCCCACTTGCGCCGTCTTTCCTTCGGCGTCAGGATGTACGTCTGGCATCCGAACACACGCATGTGCTTCACACTCGGCTTGGACTTGTAAACGATCTCGAACGGAGTCTTGTGTTCGATCTTGGGTGATGGCAGACGATTCTTCACGTAGATGGCTGTCATGGCCGCCTCAGCCCAGAAGCTCTTGTCCAGCTTGGCGTGATGCAGCATGCTGCGGCCAATCGTGACGATGGTCCGGATGGCGCGTTCTGCCGTGCCGTTGGTCTGGTGCGCGTACGGAACCGTGGTTTGCTGCTCGATGCCTTCGACTTCGTAGAAGGCCTTGAGCGAGTTGGTCGCGAACTCACGGGCTCCGTCGTGCCGCACAAACTTGACCTTCTTGCCGAACTGCGTCTGCACCTTCGTGACGTACATCTTGATGCAGTCCTCGCTCTCCGACTTGGCACGCAGGCAGAACCCCTTCATGCATCCGCTGGCTTCGTCCACGATCAGCAGCAGATACTTGCTGCCACCCAGTGACTCTTCTTCCATGGGGCCGCACACGTCGAAGTGGAGCAGCTCGAACGTGTCGTAGTGACGCTTGTCACGGTTGCTTGGGAACGGCTTCTGGACCATTTTCCCTTGCTGGCAGCCGCGACACACGCTTGGTCCACTCGACTTGGAAGGTGCCTTCGCGTCCTTGATCATGTTGTTGTCGACCATCTTGCGAAGCACCTCCACCGGAGCGTGACCCATACGCGCGTGTAAGTCGACCGTCTTCCCACTGGTCGCCGCATTCGCCGATCGCTGAGGCGTCTGGAGCCAGTAGAGTCCATCCACGAGGTTTGCCGTCGCCACCACTTGCTTGGAGCCCTTGTGCGCAATCTGCATCTTGGTGCTGTCGAACACGACTTGGAACAGACCACTCTTGTTGATCTGTGGCACCGAAAGCAGATTCTTGCTCATGCTTGGGACGTACAGCGCGTTCTTGATCTCGATGTCGCGCTCGTCGCCGTTGGGCAGAACCACTCGTTCCACGATGGTCCCCACACCCATGATGGCCGCCTTGTTGCCGTCGGCCACCGAAAGTTCGCCTTCATTCCGCTCAATCAAATGAGCGAACTTGGCCTTGTCGTTGCAGATGTGGTGCGTTGCGCCGCTGTCGACCGCCCACAACCCGGACACATTCTTGCCCGTCGAAATGCCACACTCCAGTGAGACCGCGAACGCCACTCGATCATAGCCGTAGTCGTAGTTGTAGCTGGCGTCGTCGTTCCTCCACTGGGCATTGTTGGCCCCGCGTCCACGGCCATTGCCATTGCCGCCGCGTCTTGGGCCCCGATTCTCGTCCTTCTGCTTGGTCCAGCACTTGTCAATCGTGTGCCCCAATTTCCCGCAGTACGTACACTGGCGAGACTCTCGGTCGGTGTTGAACGCCTTGGCCGCATTCTCCGTCTTCACCGTCGCCGTCTTCTCGCCTTGTCTCTTGATGTGCTCATTCGTCAGCACCTTCACGACGTCTTGCGATCGTAGTTCAGCGCTGCTCATCTCCAAGTTCAGCACGACGTTCTCGTAGCTCTTGGGGAGGCTGCGCAACAGGCAGATCGCCACGTCCTCATCCTCCATCTTGGCGCCGATGCTGCTGAGCTTGGCGCTGATGTTGAGGACCTCGTTGCAATGATGCAAGACGTTGCCGCCTTCCGACATCTCCATGCTGAAGAGTTGGCGCTTCAGGTAGATGCGTCCAGCCTTCTGCGAACCACCGTACAGCGTCTTCAGGCGCGTCCACGCAACCCACGCTTCCTCGCAGTCGTCGACCACATGGTGGTAGTCCGCGTCCATGTGGAGCAACATCAGTCCGAACGCGATGTTGCTCGACTTGACGTAGTCGTCCTTGGCCCGCGAGTCCGTGAAGCTCGGCGTCGTCTCGCGGTTCACGACATGCCACGTCGACTTGGTCAGGAACACGCCACGCATGTAGCGGTTCCACGTCGCGTAGTTGTCCCCGTTGAACTTGTCAATGGAGACCTTGGTCGAGGCGTCTTGGGCAGAGGACATGGTGCTTGGGCTCATAACCTGATGGAAGGTATGGATCTTGGACACGAAGCTCGGACGAAGCGGAGGGAGGGACGTCGCAGACGGCGGGACCGGAGCGAAGGCACTTGAGGGGTAGAATGAAGATTCGAAAGTGATTGAATTGTTGGAGCTTACACCCTACTGTTATAGACACGCGACCCTGCGTCCTATCACCTAGTGCATCTAATCAACCATCACTCACCGGTGTTCATCCAATCATCCTACTCGTCTAATCATCGGTGGCGTAAACAACTTACCGTAATCACCATAATCAACGTGGGTTCGGATTGGACCGGTTCGGGTTAACCGGTTAATGGGTACAACTGGTACACAGTACCCAAGACCAGCAACCCTCAATAGNNNGCCNTCACTNNNCTNCNNNTTACTMCATCTCAGAATGCACT

**>Psojae2019.1_contig_38_322956-327751(+)**

GGGTGCCTATTCCGACTGAAGAACAAGTATTGAGGGTTGCTGGTCTTGGGTACTGTGTACCAGTTGTACCCATTAACCGGTTAACCCGAACCGGTCCAATCCGAACCCACGTTGATTATGGTGATTACGGTAAGTTGTTTACGCCACCGATGATTAGACGAGTAGGATGATTGGATGAACACCGGTGAGTGATGGTTGATTAGATGCACTAGGTGATAGGACGCAGGGTCGCGTGTCTATAACAGTAGGGTGTAAGCTCCAACAATTCAATCACTTTCGAATCTTCATTCTACCCCTCAAGTGCCTTCGCTCCGGTCCCGCCGTCTGCGACGTCCCTCCCTCCGCTTCGTCCGAGCTTCGTGTCCAAGATCCATACCTTCCATCAGGTTATGAGCCCAAGCACCATGTCCTCTGCCCAAGACGCCTCGACCAAGGTCTCCATTGACAAGTTCAACGGGGACAACTACGCGACGTGGAACCGCTACATGCGTGGCGTGTTCCTGACCAAGTCGACGTGGCATGTCGTGAACCACGAGACGACGCCGAGCTTCACGGACTCGCGGGCCAAGGACGACTACGTCAAGTCGAGCAACATCGCGTTCGGACTGATGTTGCTCCACATGGACGCGGACTACCACCATGTGGTCGACGACTGCGAGGAAGCGTGGGTTGCGTGGACGCGCCTGAAGACGCTGTACGGTGGTTCGCAGAAGGCTGGACGCATCTACCTGAAGCGCCAACTCTTCAGCATGGAGATGTCGGAAGGCGGCAACGTCTTGCATCATTGCAACGAGGTCCTCAACATCAGCGCCAAGCTCAGCAGCATCGGCGCCAAGATGGAGGATGAGGACGTGGCGATCTGCCTGTTGCGCAGCCTCCCCAAGAGCTACGAGAACGTCGTGCTGAACTTGGAGATGAGCAGCGCTGAACTACGATCGCAAGACGTCGTGAAGGTGCTGACGAATGAGCACATCAAGAGACAAGGCGAGAAGACGGCGACGGTGAAGACGGAGAATGCGGCCAAGGCGTTCAACACCGACCGAGAGTCTCGCCAGTGTACGAACTGCGGGAAATTGGGGCACACGATTGACAAGTGCTGGACCAAGCAGAAGGACGAGAATCGGGGCCCAAGACGCGGCGGCAATGGCAATGGCCGTGGACGCGGGGCCAACAATGCCCAGTGGAGGAACGACGACGCCAGCTACAACTACGACTACGGCTATGATCGAGTGGCGTTCGCGGTCTCACTGGAGTGTGGCATTTCGACGGGCAAGAATGTGTCCGGGTTGTGGGCGGTCGACAGCGGCGCAACGCACCACATCTGCAACGACAAGGCCAAGTTCGCTCATTTGATTGAGCGGAATGAAGGCGAACTTTCGGTGGCCGACGGCAACAAGGCGGCCATCATGGGTGTGGGGACCATCGTGGAACGAGTGGTTCTGCCCAACGGCGACGAGCGCGACATCGAGATCAAGAACGCGCTGTACGTCCCAAGCATGAGCAAGAATCTGCTTTCGGTGCCACAGATCAACAAGAGTGGTCTGTTCCAAGTCGTGTTCGACAGCACCAAGATGCAGATTGCGCACAAGGGCTCCAAGCAAGTGGTGGCGACGGCAAACCTCGTGGATGGACTCTACTGGCTCCAGACGCCTCAGCGATCGGCGAATGCGGCGACCAGTGGGAAGACGGTCGACTTACACGCGCGTATGGGTCACGCTCCGGTGGAGGTGCTTCGCAAGATGGTCGACAACAACATGATCAAGGACGCGAAGGCACCTTCCAAGTCGAGTGGACCAAGCGTGTGTCGCGGCTGCCAGCAAGGGAAAATGGTCCAGAAGCCGTTCCCAAGCAACCGTGACAAGCGTCACTACGACACGTTCGAGCTGCTCCACTTCGACGTGTGCGTCCCCATGGAAGAAGAGTCACTGGGTGGCAGCAAGTATCTGCTGCTGATCATGGACGAAGCCAGCGGATGCATGAAGGGATTCTGCCTGCGTGCCAAGTCGGAGAGCGAGGACTGCATCAAGATGTACGTCACGAAGGTGCAGACGCAGTTCGGCAAGAAGGTCAAGTTTGTGCGGCACGACGGAGCCCGTGAGTTCGCGACCAACTCGCTCAAGGCCTTCTACGAAGTCG**AAGGCATCGAGCAGCAAACCACGGTTCCGTACGCGCACCAGACCAACGGCACGGCAGAACGCGCCATCCGGACCATCGTCACGATTGGCCGCAGCATGCTGCATCACGCCAAGCTGGACAAGAGCTTCTGGGCTGAGGCGGCCATGACAGCCATCTACGTGAAGAATCGTCTGCCATCACCCAAGATCGAACACAAGACTCCGTTCGAGATCGTTTACAAGTCCAAGCCGAGTGTGAAGCACATGCGTGTGTTCGGATGCCAGACGTACATCCTGACGCCGAAGGAAAGACGGCGCAAGTGGGACCCCAAGGCTCGCGCCGGGCTGTTCCTCGGCTACGAAGAAGTGTCCAAGGCGTATCGAGTTTATGACATCGAGGCGGGGCAGGTGGTTATCAGTCGCGATGTCAACTTCGATGAGTCGACCTTTGGACTTTCGACGCATACTTCGGATGAAGACGTCGATGATGCTGTGCTGGACTTGGACGCGCTGGACCTGGACGACAACGATGGACCGCGCCAAACGAGCTTCAAGCAGGCGGGGAAGCGCAAGTACCGACCGAGTGGCGACGACAACACTGCCAGAAGATCGCGCCCCGTTCGCCACCGAGCTGGCTTAGAAGAAGCAAGTGCGCCGGACGTATCCTCCTCGCGCCGAGCCGACCCCGACGAAGAAGAAAAGTCTGGGGACCAAGATGAAGACGACGACTCGACGCCTCCAGTGTTTTGGCGTGCCAGTGCCAACGCTGTGGAAGCAGCAGCCGATCTATCGGAGCCGACGACGTTCCAAGAGGCTGTGAACGGTCCTGATCAAGTGCACTGGCGCAAAGCGATCCGCGCGGAGCTCAAGTCGATGCGACTGCGTGGTGTGTTTCGAGCTGCCAAGTTGCCTAACGGACAACGCGCCATCGATACCAAGTGGGTGTTCAAGATCAAGCGCAAAGCGGACGGCTCGATCGAGAAGTACAAGGCGCCCCTCGTGGCCAAGGGCTTCAAGCAGAAGTACGGCATTGACTACACCGAGACGTTCTCGCCCGTGGTCAAGT**ATGTGACGCTGCGCATGGTGATCGCGATTGCTAAGTATTTTGGCTGGCCCCTCGACCAACTCGACGTGGTGACAGCATTCCTGTACGGGATAATGAAGGAGGTGGTCTTCTGCGTGGTTCCGGAAGGCGTCGAACTGGATGGTGACTTCGACTGCCTGGAACTGGTGAAGGCCATCTACGGTCTCAAGCAAGCTTCGCGAGTGTGGAACGAGACCTTCGACGAGTTCGTGTGCTCCATCGGCTTCCAAGTGTCGGCCTTCGATCCGTGCCTCTACATTAAGGTGGTGGACGGTCACTGCGTGCTTGTACTGGTGTACGTGGATGACGTACTGATTACCAGGAGTTCGCCCGAGCTGATCGCACGCACGAAGACCGACCTGAAGACGCGCTTCGAGATGACCGACAGCGGCAAGTGCGCGTTTGTTCTTGGGATCGAGTTGGTGGACGGCCCGGACGGCAGCGTGACCATGTGCCAGCGGCGATACGTGGACGACATCCTGAAGCGCTTCGCCATGGACGAGTGCAAGGCCGTGGTGAGTCCGGTGGACATGAGCACTCGACTGGTTCCAAGCGACGCGGCGACCAAGGTGAACGCTCCGTTTCGCGAAGCTGTTGGTGCTCTGATGCACCTGATGACCGCCACGCGCCCAGACATCGCGTACGCCGTTGGCTATGTATCGCGCTTCATGGAGAACCCCCAAGAGGAACACTGGGTTGCGGTCAAGCGTATCTTTCGCTACCTGCAAGGCACCAAGACGCATGGGATCTGCTTCAAGCCGGGCGACAAGATCGACTTTCGTGGCTACTCGGACGCGGACTGGGCCGGCGACCTCGCCGACCGCAAGTCCACTTCAGGCTACACGTTCATGCTCATGAGTACGCCTGTGAGCTGGGGAAGCAAGAAGCAATCAAGTGTGTCGCTGTCCACGAGCGAAGCGGAGTACATCACGCTCAGTCTGGCTATCCAAGAAGGCAAGTGGATTCATCGCCTGCCATGCGAGATTCTGGCGGCCGCTAATGAGACTGGACCCGAACTCATGATCCGCGAAGACAACCAGTCGTGCATCAAGATGACGAAGAACCCAGTGAGCCACGGTCGCGCCAAGCACATCGACATCAAGTACCACCACATCCGCGATGAAGTCAAGCGCGGCGAAGTCAAGCTCCAGTACTGCGAGACTTCTGTGATGCTAGCGGACATCATGACGAAGGCGCTTGCGGGACCACGCCACACGGATCTGACGGTGGCTCTCGGCATTCGTGGGACTTCGCATTGAGGGGGCGTATTGAGGGTTGCTGGTCTTGGGTACTGTGTACCAGTTGTACCCATTAACCGGTTAACCCGAACCGGTCCAATCCGAACCCACGTTGATTATGGTGATTACGGTAAGTTGTTTACGCCACCGATGATTAGACGAGTAGGATGATTGGATGAACACCGGTGAGTGATGGTTGATTAGATGCACTAGGTGATAGGACGCAGGGTCGCGTGTCTATAACAGTAGGGTGTAAGCTCCAACAATTCAATCACTTTCGAATCTTCATTCTACCCCTCAAGTGCCTTCGCTCTGGTCCCGCCGTCTGCGACGTCCCTCCCTCCGCTTCGTCAGAGCTTCGTGTCCAAGATCCATACCTTCCATCAGCGACAATAGATTCGCAAGCTGATCGC

ATG – predicted Start codon

TAG – predicted stop codon

The LTR marks (5’-TG..CA-3’) are underlined within the LTR regions highlighted in cyan.

**Nucleotides in bold**: The probe (GenBank: JF519821.1) used for fingerprinting of *P. sojae*

Nucleotides underlined: The probe (GU244585.1) used for fingerprinting of *P. sojae*

Alignment of the Left and Right LTRs

Left TGAGGGTTGCTGGTCTTGGGTACTGTGTACCAGTTGTACCCATTAACCGGTTAACCCGAA 60

Right TGAGGGTTGCTGGTCTTGGGTACTGTGTACCAGTTGTACCCATTAACCGGTTAACCCGAA 60

************************************************************

Left CCGGTCCAATCCGAACCCACGTTGATTATGGTGATTACGGTAAGTTGTTTACGCCACCGA 120

Right CCGGTCCAATCCGAACCCACGTTGATTATGGTGATTACGGTAAGTTGTTTACGCCACCGA 120

************************************************************

Left TGATTAGACGAGTAGGATGATTGGATGAACACCGGTGAGTGATGGTTGATTAGATGCACT 180

Right TGATTAGACGAGTAGGATGATTGGATGAACACCGGTGAGTGATGGTTGATTAGATGCACT 180

************************************************************

Left AGGTGATAGGACGCAGGGTCGCGTGTCTATAACAGTAGGGTGTAAGCTCCAACAATTCAA 240

Right AGGTGATAGGACGCAGGGTCGCGTGTCTATAACAGTAGGGTGTAAGCTCCAACAATTCAA 240

************************************************************

Left TCACTTTCGAATCTTCATTCTACCCCTCAAGTGCCTTCGCTCCGGTCCCGCCGTCTGCGA 300

Right TCACTTTCGAATCTTCATTCTACCCCTCAAGTGCCTTCGCTCTGGTCCCGCCGTCTGCGA 300

****************************************** *****************

Left CGTCCCTCCCTCCGCTTCGTCCGAGCTTCGTGTCCAAGATCCATACCTTCCATCA 355

Right CGTCCCTCCCTCCGCTTCGTCAGAGCTTCGTGTCCAAGATCCATACCTTCCATCA 355

********************* *********************************

**>** **Psojae2019.1_contig_38_322956-327751(+)**

MSPSTMSSAQDASTKVSIDKFNGDNYATWNRYMRGVFLTKSTWHVVNHETTPSFTDSRAKDDYVKSSNIAFGLMLLHMDADYHHVVDDCEEAWVAWTRLKTLYGGSQKAGRIYLKRQLFSMEMSEGGNVLHHCNEVLNISAKLSSIGAKMEDEDVAICLLRSLPKSYENVVLNLEMSSAELRSQDVVKVLTNEHIKRQGEKTATVKTENAAKAFNTDRESRQCTNCGKLGHTIDKCWTKQKDENRGPRRGGNGNGRGRGANNAQWRNDDASYNYDYGYDRVAFAVSLECGISTGKNVSGLWAVDSGATHHICNDKAKFAHLIERNEGELSVADGNKAAIMGVGTIVERVVLPNGDERDIEIKNALYVPSMSKNLLSVPQINKSGLFQVVFDSTKMQIAHKGSKQVVATANLVDGLYWLQTPQRSANAATSGKTVDLHARMGHAPVEVLRKMVDNNMIKDAKAPSKSSGPSVCRGCQQGKMVQKPFPSNRDKRHYDTFELLHFDVCVPMEEESLGGSKYLLLIMDEASGCMKGFCLRAKSESEDCIKMYVTKVQTQFGKKVKFVRHDGAREFATNSLKAFYEVEGIEQQTTVPYAHQTNGTAERAIRTIVTIGRSMLHHAKLDKSFWAEAAMTAIYVKNRLPSPKIEHKTPFEIVYKSKPSVKHMRVFGCQTYILTPKERRRKWDPKARAGLFLGYEEVSKAYRVYDIEAGQVVISRDVNFDESTFGLSTHTSDEDVDDAVLDLDALDLDDNDGPRQTSFKQAGKRKYRPSGDDNTARRSRPVRHRAGLEEASAPDVSSSRRADPDEEEKSGDQDEDDDSTPPVFWRASANAVEAAADLSEPTTFQEAVNGPDQVHWRKAIRAELKSMRLRGVFRAAKLPNGQRAIDTKWVFKIKRKADGSIEKYKAPLVAKGFKQKYGIDYTETFSPVVKYVTLRMVIAIAKYFGWPLDQLDVVTAFLYGIMKEVVFCVVPEGVELDGDFDCLELVKAIYGLKQASRVWNETFDEFVCSIGFQVSAFDPCLYIKVVDGHCVLVLVYVDDVLITRSSPELIARTKTDLKTRFEMTDSGKCAFVLGIELVDGPDGSVTMCQRRYVDDILKRFAMDECKAVVSPVDMSTRLVPSDAATKVNAPFREAVGALMHLMTATRPDIAYAVGYVSRFMENPQEEHWVAVKRIFRYLQGTKTHGICFKPGDKIDFRGYSDADWAGDLADRKSTSGYTFMLMSTPVSWGSKKQSSVSLSTSEAEYITLSLAIQEGKWIHRLPCEILAAANETGPELMIREDNQSCIKMTKNPVSHGRAKHIDIKYHHIRDEVKRGEVKLQYCETSVMLADIMTKALAGPRHTDLTVALGIRGTSH*

GAG, Capsid protein (pfam14223)

PR, GAG-pre-integrase (pfam13976)

INT, Integrase (pfam00665)

RT, Reverse transcriptase (pfam07727)

RH, RNase H (cd09272)

Domains are annotated based on CD-search, hereafter.

**>Bremia_lactucae_Sca7_2388-7076(+)**

ATGTCTTGCGTCGAAGAGTATTGAGGGTTATGGGTTTCAGGTAATGTACACAACCCAAACCGGTCCAATCCGAACCGCGGTTAAACATAGCATCTACTTATATTATAGTATAGTTAGATGCAGTATGTAGAAATGAGTGGTTTCGAGAAGTAAAAGTGTAAGCCCCAATAAATCAATCACTTCCAAATATTCCTTCTCCTTCACAAGTCGTCTTCACCTTTCAAGTGCAGACTTCTCTCGTGTTCTTCAAGCCTCCTTCTCTCTTCCAGTGCTCGGTACCCTTTAACAGGTTATGAGCCCAAGCACGATGTCCTCTGCTCAAGATTCCGCCACCAAGATCTCCATCGACAAATTCGATGGCGACAACTATGCGACGTGGAGCCGCTACATGCGTGGCGTGTTCCTGACCAAGTCGACTTGGCATGTGGTCAACCGGGAGACTACTCCAACCTTCGCCGATCCTCGCGCCATGGACGAATATGTCAAGGCGAGCAATATCGCCTTCGGCTTGATGTTGCTGTACATAAGCGCGGATTACCATCACGTGGTTGATGACTGCGAAGAAGCATGGGTTGCGTGGGCAAAGCTGAAGACCCTCTACGGCGGATCGCAGAAGGCAGGACGGATCTTCTTGAAGCGACAGCTCTTCAGCATGGAGATGGCGGAAGGCGGTAATGTACTTCATCATTGCAATGAAGTCCTCAACATCAGCGCAAAGCTCAAGAGCATCGGCGCCAAGATGGAGGACGAGGACGTGGCGATTTGCTTGTTGCGCAGCCTCCCCAAGAGTTACGAGAACGTCGTTCTCAACTTGGAGATGAGCAGCGCGGAATTGCGATCGCGGGACGTCGTGAAAGTGCTGACAAATGAGCACATCAAGAGACAAGGAAACAAGACGACATCGGTGAAGACTGAAGAAACAACCAAGGCTTTCAGTACCGAACGCGAGTTTCGTCAGTGTACATTCTGCGGAAAATTGGGGCACACTGTGGAGAAGTGCTGGACCAAGCAGAAGGAAGACAATCGGGGAGCTCGACGCGGCGGCAATTATCGTGGACGCGGAGCGAATCAAGTTCAGTGGCAAGGCTACAGTGGCAACAGCAACGGTAACTACGATCGTGTGGCGTTTGCTGTGTCAATGAAATGCGGACTTTCAACGACCAAGAACATGTCCGGGATGTGGGCGATTGATAGCGGTGCTACGCATCACATCTGCTACGAAAAATCCAAGTTCGAAGTTCTGGACGAACACAATGAAGGTGAAGTTCTGGTCGCAGATGGAAATAAAGCCGCTATCAAAGGCATCGGGACTATCATCGAAAAGGTACTACTGCCTAACGGTGAAGAACGTGAAGTCGAGATCAAAAACGCGCTTTTCGTCCCAGATATGAACAAGAACCTACTATCAATTCCGCAAATCAACAAGAGCGGCAAGTTTCAAGTGGTGTTTGATGGTACCAAAATGGAGATCTCGCGTAAAGACCCTGAGCAAGTGGTGGCGATGGCAGATCTTGCGGATGGACTCTATTGGCTTCGCACATCTCACCGATCGATAAATACTACATCAAGGTCAAGAGTCGAAGATCTTCATGCACGCATGGGCCACGCACCGTTTGATGTCTTGTGCAAAATGGTTTCCAAAGGGATGATCAAGGATGCTAAAATGCCAGAAAAGGCAAGCAGATCTAGCATATGCCACGGGTGTCAAGAAGGAAAAATGGTACAGCGACCGTTTCCAAGTAACCCGAACAAGCAGCACTACGACCCGTTCGAGCTTCTACACATCGACACTTGCGGACCCATGGAAGTTGAATCGCTGGGTGGAAGCAAGTACTTGCTACTAATCGTCGATGAAGGCAGCGGATGCATGAAGGGCTTCGGTCTGCGCGCCAAGTCTGATAGTGAAGAGTGTATCAAGAGATACATCAATGCGGTGCAGACGCAGTTTGACTTCAAGGTCAAGTTCGTTCGACATGATGGTGCACGAGAATTCGCGACAACTTCGATCAAGGCATTTTACGATGATCAAGGAATCGAGCAGCAAGTCACTGTTCCGTATGCGCACCAGACGAATGGCACGGCAGAACGGGCAATCCGGACTATCGTAACGATTGGCCGCAGCATGCTCCACTACGCAAAATTAGACAAGTGTTTCTGGGCTGAGGCAGCAATGACAGCAATATACATCAAGAATCGACTGCCATCGCCCAAGTACCAAGATCAGACTCCGTTCGAGATCATCAACGGATCTCGACCAAGTGTCAAACACATGCGAGTTTTTGGCTGCCGTGCGTTCGTGCTGACTCCAAAAGAAAAGAGATCGAAGTGGGATCCGAAGTCCCGTGAAGGACTGTTTATGGGGTACGAAGAAGCATCAAAGGCATACCGCATTTACGACATTGAAACGGATCAGGTGGTGATATCTCGCGATGTCACGTTCGACGAATCGACGTTTGGGTTCGCACAGACGCCTCTACAAGATGTTGTTGATGATACCTTATTGGATTTTGACACGATGAGCATCAGTAGTGAACCTGCCACCACGGAGTTCAAGCAAACAGGCAAGCGCAAGAACCGTTCAAACAGCCAAGAACAAGTGTTTAAACGACCAGCTTGCCGCGGAGCCGGGTTAGAAGAAGCAAGTGCGCCGGATGACTTCGAGTTGCGCCACCAAAAACGACGTTCGAATGTTCGAGCTAACCGGGACGAAGAGCAAAAGGACAATGATATGGATGATGATGACGACACCCACCACGGTTTTTGGCAAGCAAGCGTGAATGCAGTTGAGGGCGCTGATTTATCCGAACCAACAACGTTTGAAGACGCGATTAATGGGCCAGATCGAGTACACTGGCGTAAGGCGATCTGTGCGGAGCTAGACTCAATGAAACTCCGTAGCGTATTTCGAGCAACGAAGCTGCCAGCTGGGCAGCACACCATTGGGACCAAGTGGGTATTCAAGATCAAGCGCAAGGCTGACGGAAACATCGATAAATACAAGGCACGTCTCGTTGCCAAGGGTTTCAAGCAAAAGTATGGCATCGACTACACGGAGACGTTTTCACCAGTAGTCAAGTGCGTGACGCTGCGCATGGTGATTGCACTCACAAAGTACTTCGACTGGACACTGGACCAACTGGACGTGGTGACAGCTTTTCTGTATGGAGAAATGAAGGAAAGAGTATTCTGTGCGGTCCCAGAAGGAGTCGACGTCAATAAAGATTTTGATTGCTTTGAACTGGTTAAAGCAATCTACGGACTAAAACAAGCTTCACGGGTATGGAATGACACTTTTCATGAGTTTGTTTGCTCCATCGGATTTCAAGTCTCCGATTTTGATCCGTGCCTTTATCTCAAGATTGCAAACGGAGAGTGCGTGCTATTGTTGGTCTATGTTGATGATGTACTGGTGACTGGGAGTTCAATTGAAATGATTGCGCACACCAAGAAAGACTTAAAGTCACGTTTTGAGATGACTGATAGTGGCAAGTGCGCTTTTGTCCTCGGCATCGAGCTGGTAGACAACAATGACGGAAGCGTGACGATGTGCCAGCGACGCTACGTGGACGATGTTCTCAAGCGTTTCGGCATGAGTGACTGCAAAGCTGTCATCAGCCCAATGGACATCAGCTCTCGACTAACGTCAAGCAACGCAGCAACTAAAATCAACGTTCCGTTTCGTGAAGCAGTGGGTGCTTTAATGCACTTGATGACCGCGACACGACCAGACATTGCCTTCGCCGTGGGTTACGTGTCGCGTTTTATGGAAAACCCACAAGTCGAGCATTGGATGGCGGTGAAGCGCATCCTTCGATACTTGCAAGGAACCAAGTCGCATGGAATCTGCTTTAAGCCTGGCAACAATGTGGATTTTTGCGGCTACTCGGATGCAGACTGGGCCGGTGATCATGCAGACCGCAAGTCGACTTCAGGATACGCGTTTATTCTCATGGGCGCTCCTGTAAGCTGGGGGAGCAAGAAACAATCAAGTGTGTCGCTGTCAACAAGTGAAGCCGAATATATCGCGCTAAGTTTAGCGATTCAAGAAGGCAAGTGGGTGCATCGACTACTGCGCGAAATCCTAGACGCGGCAGCAAACAAGTCTGAACCGGTGCTTAAGATCATGGAAGACAACCAGTCGTGTATCAAGATGACGAAGAATCCGGTAAACCATGGTCGTGCGAAACATATCGACATCAAGTATCACCACATTCGTGATGAAGTAAAGCGTGGTGATGTCAAGTTGGAATACTGCGAGACTTCGAAGATGTTGGCGGATATCATGACGAAAGGACTGCCAGGACCGCGTCACAAGGAATTAACGACAGCGCTTGGCATACACGCGTGTTAGCATTGAGGGGGCGTATTGAGGGTTATGGGTTTCAGGTAATGTACACAACCCAAACCGGTCCAATCCGAACCGCGGTTAAACATAGCATCTACTTATATTATAGTATAGTTAGATGCAGTATGTAGAAATGAGTGGTTTCGAGAAGTAAAAGTGTAAGCCCCAATAAATCAATCACTTCCAAATATTCCTTCTCCTTCACAAGTCGTCTTCACCTTTCAAGTGCAGACTTCTCTCGTGTTCTTCAAGCCTCCTTCTCTCTTCCAGTGCTCGGTACCCTTTAACAAAGCCCCGTCATGCCTCACAAACTTGACTTTTTCCGAACTGCGTTAGGACCTTCA

ATG – predicted Start codon

TAG – predicted stop codon

TGA – The corresponding predicted stop codon in *P. sojae*

The LTR marks (5’-TG..CA-3’) are underlined within the LTR regions highlighted in cyan.

**>P_citricola_Sca60_26953-31785(-) (predicted coding region is truncated)**

GGGTGAACTTCCGGTAATCAGTATTGAGGGTTGCTGGTCTTGGGTACTGTGTACCAGTTGTACCCATTAACCGGTCAACCCGAACCGGTCCAATACGAACCCACGTTGATTATGGTGATTACGGTAAGTTGTTTACGCCACCGATGATTAGACGAGTAGGATGATTGGACGAACACCGATGAGTGATGGTTGATTAGATGCACTAGGTGATAGGACGCAGGGTCGCGTGTCTATAACAGTAGGGTGTAAGCTCCAACAATTCAATCACTTTCGAATCTTCATTCTACCCCTCAAGTGTCTTCGCTCCGGTCCCGCCGTCTGCGACGTCCCTCCCTCCGCTTCGTCCAAGCTTCGTGTCCAAGATCTATACCTTCCATCAGGTTATGAGCCCAAGCACCATGTCCTCTGCGCAAGACGCCTCGACCAAGGTCTCCATTGACAAGTTCAACGGGGACAACTACGCGACGTGGAACCGCTACATGCGTGGCGTGTTCCTGACCAAGTCGACGTGGCATGTGGTGAACCGCGAGACGACGCTGAGCTTCACGGACTCGCGGGCCAAGGACGAGTACATCAAGTCGAGCAACATCGCGTTCGGACTGATGTTGCTCCACATGGACGCGGACTACCACCACGTGGTCGACGACTGCGAGGAAGCGTGGGTTGCGTGGACGCGCCTGAAGACGCTGTACGGTGGTTCGCAGAAGGCTGGTCGCATCTACCTGAAGCGCCAACTCTTCAGCATGGAGATGTCGGAAGGCGGCAATGTGTTGCATCATTGCAATGAGGTCTCAACATCAGCGCCAAGCTCAGCAGCATCGGCGCCAAGATGGAGGACGAGGACGTGGCGATCTGCCTGTTGCGCAGTCTCCCCAAGAGCTACGAGAACGTCGTGCTGAACTTGGAGATGAGCAGCGCTGAACTGCGGTCGCAAGACGTCGTGAAGATGCTCACGAATGAGCACATCAAGCGGCAAGGCGAGAAGACGGCGACGGTGAAGACGGAGAACGCGGCCAAGGCGTTCAACACCGACCGAGAGTCTCGCCAGTGCACGTACTGCGGTAAATTAGGGCAAACGGTGGACAAGTGCTGGACCAAGCAGAAGGACGAGAATCGGGGCCCAAGACGCGGCGGCAATGGTAATGGCCGTGGACGCGGGGCCAACAACATCCAGTGGAGAAACGACAACGACGGCTACGACTACGACTACGACTACGATCGAGTGGCGTTCGCGGTTTCCATGGAATGTGGTATTCCGACGGGCAAGAATGTGTCCGGGATGTGGGCGGTCGACAGCGGCGCAACGCACCACATCTGCAACGACAAGGCCAAGTTCGCCTATTTGATCGAGCGAAACGAGGGCGAACTTTCGGTGGCCGACGGCAACAAGGCGGCCATCATGGGTGTGGGGACCATCGTGGAACGAGTGGTTCTGCCCAACGGCGACGAGCGCGAGATCGAGATCAAGAACGCGCTGTACGTGCCAAGCATGAGCAAGAATCTGCTTTCGGTACCGCAGATCAACAAAAGTGGCAAGTTCCAAGTGGTGTTTGACGGCGCCAAGATGCAGATTGCGCGCAAGGACTCCAAGCAAGTAGTGGCGACGGCAAACCTCGTGGATGGACTCTACTGGCTGATGACGCCTCAGCGATCGGCGAATGCGGCGACGAGTGGCAAGATTGTCGACCTGCACGCGCGCATGGGTCACGCTCCGGTTGAGGTGCTTCGCAAGATGGTCGACAACAACATGATCAAGACGCGAAGGCACCTTCCAAGTCGAGCGGACCAAGTCTGTGTCGTGGTTGCCAGCAAGGGAAGATGGTCCAAAACCGTTCCCAAGCAACCGTGACAAGCGCCACTACGACACCTTCGAGTTGCTCCACTTCGACATCTGCGGACCCATGGAGAAAAACTCGCTTGGCGGCAGCAAGTACCTGCTGCTGATCGTGGACGAAGCCAGCGGATGCATGAAGGGTTTCTGCCTGCGTGCCAAGTCGGAGAGCGAAGACTGCATCAAGACGTACATCATGAAGGTGCAGAAGCAGTTCGGCAAGAAGGTCAAGTTTGTGCGGCACGACGGAGCTCGTGAGTTTGCGACTAACTCGCTCAAGGATTTCTACGAAGACGAAGGCATCGAGCAGCAAACCACGGTCCCGTACGCGCACCAGACCAACGGCACGGCAGAGCGAGCTATCCGGACTATCGTCACGATCGGCCGCAGCATGCTGCATCACGCTAAGCTGGACAAGTGTTTCTGGGCTGAGGCGGCAATGACGGCCATCTACGTGAAGAACCGTCTGCCGTCACCCAAGATCGAGCACAAGACTCCGTTCGAGATCGTGTACAAGTCCAAGCCAAGTGTGAAGCACATGCGTGTGTTCGGCTGTCGGACGTACATTCTGACTCCGAAGGAGAAGCGGCTCAAGTGGGATCCCAAGGCTCGCGCCGGATTGTTTCTCGGCTACGAAGAAGTGTCCAAGGCGTATCGAGTTTATGACATCGAGGCGGGGCAGGTGGTCATCAGTCGCGATGTCAACTTCGATGAGTCGGCCTTTGGACTTTCGCCGCAGATTTCCGATGAAGATGTCGATGACCTGGACTTCGACTCGCTCGACATCGACGATGACGGTCCTCGCCAGACGGAATACAAGCAGGCGGGGAAACGCAAGAGTCGCCCAAGTGACGAAGATGAAGGTACCAGAAGACCGCGCACTGTGCGTCATCGGCCTGGATTGGAAGAAGCAAGTGCGCCGGACTCCTCTTCGCACCGAGTGGACACCAACGAAGAAGAAAAGTCTGGTGACCAAGATGAAGAATCGACTTCTCCTGTGTTTTGGCGTGCCAGTGCCAACGCTGTCGAAGCAGCAGCCGATCTATCGGACCCGTCGACATTCCAAGAAGCGGTGAACGGTCTGATCAAGTGCACTGGCGCAAGGCTATTCGTGCGGAGCTCAAGTCGATGCGACTTCGTGGTGTGTTTCGTGCTGCCAAGTTGCCTAACGGACAACGCGCCATCGGCACCAAGTGGGTGTTCAAGATCAAGCGCAAAGCGGACGGATCGATCGAGAAGTACAAGGCGCGCCTCGTGGCCAAGGGGTTCAAGCAGAAGTACGGCATCGACTACACGGAGACGTTCTCGCCCGTCGTCAAGTACGTGACGCTGCGCATGGTGATCGCGATTGCCAAGTATTTTGGCTGGCCCCTCGACCAACTCGACGTGGTGACTGCATTCCTGTACGGGATAATGAAGGAGCTGGTGTTCTGCGCGGTCCCCGAAGGTGTTGATCTCGACGGTGACTTCGACTGCCTGGAATTGGTGAAGGCGATCTACGGTCTCAAGCAAGCTTCGCGCGTGTGGAACGAGACCTTCGACGAGTTCGTGTGCTCCATCGGCTTCCAAGTATCGGCGTTCGACCCATGCCTCTACATCAAGATCGTTGACGGTCACTGCGTGCTCGTGCTGGTCTACGTGGATGACGTGCTGATTACCGGAAGTTCACCCGAACTGATCTCACGCACGAAGACCGACCTCAAGACACGCTTCGAGATGACCGACAGCGGCAAGTGCGCGTTCGTTCTTGGGATCGAGTTGGTGGACGGCCCGGACGGCAGCGTGACGATGTGTCAGCGCCGCTATGTGGATGACATTCTCAAGCGTTTTGGTATGGACGAGTGCAAGGCCGTGGTGAGTCCCGTCGACATAAGCACTCGACTGATTTCAAGTGACGCAGCGACCAAGGTGAACGCTCCGTTTCGCGAAGCTGTTGGTGCTCTGATGCACTTGATGACTGCCACGCGCCCGGACATCGCGTACGCCGTTGGCTATGTGTCGCGCTTCATGGAGAACCCTCAAGAAGAACACTGGGTTGCGGTCAAGCGTATCTTTCGCTACCTGCAAGGCACCAAGACGCATGGCATCTGCTTCAAGCCGGGTGACAACATCGACTTTCGTGGATACTCGGACGCTGACTGGGCTGGCGACCTCGCGGACCGCAAGTCGACTTCCGGCTACACGTTCATGCTGATGAGTGCGCCTGTGAGCTGGGGAAGCAAGAAGCAGTCAAGTGTGTCGCTGTCTACGAGCGAGGCGGAGTACATCGCGCTCAGTCTGGCTATCCAAGAGGGCAAGTGGATCCATCGCCTACTGTGCGAGATTTTGGCGGCTACCAACGAGACGGGACCCGAGCTCATGATCCGCGAAGACAACCAGTCGTGCATCAAGATGACGAAGAACCCAGTGAACCACGGTCGCGCCAAGCACATCGACATCAAGTACCACCACATCCGCGACGAAGTGAAACGCGGTGAAGTGAAGCTCGAGTATTGCGAGACTTCTATGATGCTGGCGGACATCATGACGAAGGCGCTCCGGGACCTCGTCACACGGACCTGACGGCTGCTCTCGGCATCCACGCGTGTTCGCATTGAGGGGCGTATTGAGGGTTGCTGGTCTTGGGTACTGTGTACCAGTTGTACCCATTAACCGGTCAACCCGAACCGGTCCAATACGAACCCACGTTGATTATGGTGATTACGGTAAGTTGTTTACGCCACCGATGATTAGACGAGTAGGATGATTGGACGAACACCGATGAGTGATGGTTGATTAGATGCACTAGGTGATAGGACGCAGGGTCGCGTGTCTATAACAGTAGGGTGTAAGCTCCAACAATTCAATCACTTTCGAATCTTCATTCTACCCCTCAAGTGTCTTCGCTCCGGTCCCGCCGTCTGCGACGTCCCTCCCTCCGCTTCGTCCAAGCTTCGTGTCCAAGATCTATACCTTCCATCAATCAGCACGTCATCCACGTAGACCAGCACGAGCACACAGTG

ATG – predicted start codon

TAG – predicted stop codon

The LTR marks (5’-TG..CA-3’) are underlined within the LTR regions highlighted in cyan.
